# Recursion beyond language: Lexical and arithmetic interference in visual hierarchical embedding

**DOI:** 10.1007/s00426-026-02252-2

**Published:** 2026-03-10

**Authors:** Mauricio J.D. Martins, Daniel J. Cook, Arno Villringer

**Affiliations:** 1 SCAN-Unit, Department of Cognition, Emotion, and Methods in Psychology, Faculty of Psychology, University of Vienna, Vienna, Austria; 2 Technology Infrastructure Department, Site Reliability Engineering Group, Major League Baseball, New York, NY USA; 3 Berlin School of Mind and Brain, Humboldt Universität zu Berlin, Berlin, Germany; 4 Max Planck Institute for Human Cognitive and Brain Sciences, Leipzig, Germany; 5 Clinic for Cognitive Neurology, University Hospital Leipzig, Leipzig, Germany

## Abstract

The capacity to represent recursive hierarchical embedding (RHE) is considered a hallmark of human cognition. Yet, it remains debated whether non-linguistic recursion depends on language-specific, domain-general, or independent visuospatial mechanisms. In two experiments, we tested this question using a dual-task paradigm. Participants performed either a visual recursion task (REC) or a matched visual iteration task (ITE) while concurrently engaging in lexical retrieval (LEX), serial arithmetic (MATH), visual delayed match-to-sample (VIS), or no interference (NONE). Both LEX and MATH—tasks that require serial, symbolic, generative processing—reliably reduced accuracy in REC and ITE, with interference being stronger for ITE. Drift diffusion modeling confirmed that LEX and MATH slowed evidence accumulation and reduced decision thresholds, indicating less efficient and more impulsive responding. Interestingly, while LEX and MATH lengthened response times in ITE, they shortened them in REC, suggesting compensatory processing strategies. By contrast, VIS did not impair accuracy; instead, it increased drift and boundary separation, consistent with more cautious evidence accumulation. Cognitive covariates further revealed that verbal working memory predicted REC more strongly than ITE under baseline conditions; however, this advantage did not account for the greater interference observed in ITE. Taken together, these findings suggest that visual hierarchical processing involves serial symbolic resources, but recursion is more resilient than iteration, possibly due to the availability of parallel or configural strategies. This supports a hybrid architecture in which linguistic-like symbolic operations contribute to building visual hierarchies, but alternative mechanisms can buffer recursion when these resources are taxed.

**Supplementary Information:**

The online version contains supplementary material available at 10.1007/s00426-026-02252-2.

## Significance

Recursive structure is fundamental to human cognition, yet its cognitive basis outside language is debated. Using an interference paradigm, we show that visual hierarchy building depends on symbolic resources tapped by lexical retrieval and serial arithmetic, while purely visual load does not impair accuracy. Iteration is more vulnerable than recursion, suggesting it is less able to recruit compensatory configural-parallel mechanisms. These findings refine theories of hierarchical cognition and offer a general framework for testing recursion across domains beyond language.

**Supplementary Information:**

The online version contains supplementary material available at 10.1007/s00426-026-02252-2.

## Highlights

Developed a dual-task paradigm to assess the cognitive bases of visual hierarchical processing.

Lexical retrieval and serial arithmetic impaired both visual recursion and iteration, whereas visual interference did not.

Drift diffusion modeling showed that symbolic interference reduced evidence accumulation and lowered decision thresholds.

Under interference, visual recursion shortened response times while outperforming iteration in accuracy.

Findings suggest visual recursion primarily relies on serial symbolic generative systems but flexibly compensates when taxed.

**Supplementary Information:**

The online version contains supplementary material available at 10.1007/s00426-026-02252-2.

## Introduction

The capacity to generate and represent multilayered hierarchical structures is a hallmark of human cognition. Humans can create complex hierarchies in various domains, including language, music, vision, action sequencing, and the social domain (Fitch & Martins, 2014; Hauser et al., 2002; Jackendoff & Lerdahl, 2006; Koechlin & Jubault, 2006; Koelsch et al., 2013; Kumaran et al., 2016; Martins, 2012; Rohrmeier, 2011). However, the cognitive mechanisms underlying this exceptional generative capacity in humans are a source of debate (Berwick & Chomsky, 2016; Fedorenko & Shain, 2021; Hauser et al., 2002; Jackendoff & Pinker, 2005).

Recursive hierarchical embedding (RHE) is one of the core capacities thought to contribute to this generative power (Chomsky, 2007; Friederici et al., 2017; Hauser et al., 2002; Martins, 2024). In formal sciences, recursion describes a kind of function in which an element category appears on both sides of a transformation rule (Fitch, 2010; Odifreddi, 1992). For example, repeatedly applying the function N_(i)_ = N_(i-1)_ + 1 generates the infinite set of natural numbers {1, 2, 3,…}. While powerful, recursion by itself does not create hierarchies (Fitch, 2010; Lobina, 2011; Martins, 2012). Additionally, hierarchical embedding is the process by which asymmetrical dependencies are generated between objects, specified by a system of rules. For example, with the rules A → A[B] and B → B[C], we could create the structures A[B], B[C], A[B[C]], or even structures with an infinite set of objects like A[BBBBBBBBB…]. However, this generative system cannot create more depth than the number of hierarchical rules. Together, recursion and hierarchical embedding allow objects to be combined in an unbounded hierarchical fashion. For example, the repeated application of the rule A → [[A] A] can create the unbounded hierarchy [[[A] A] A …].

In language, there are several examples of this recursive hierarchical embeddedness. We can generate compound nouns by combining noun phrases, NP → [[NP]NP], such as embedding [student] into [committee] to form [[student] committee]. This is a type of committee, not a type of student. Repeating the operation could further extend the hierarchical depth, e.g., [[[medical] student] committee]. Importantly, not all linguistic hierarchical structures occur between elements of the same category, and much of the recursion in language is indirect, involving the combination of more than one rule (Berwick & Chomsky, 2016; Roeper, 2011). For example, the two-rule system specifies how sentences (S), noun phrases (NP), verb phrases (VP), and verbs (V) can be combined:

S → [[NP] [VP]]

VP → [V [that [S]]]

With these two rules, we can embed sentences in verb phrases, which are embedded in sentences, thus allowing unbounded hierarchical depth. For example:

1. [[Ben]_NP_ [left]_VP_]]_S_.
2. [[Anna]_NP_ [[thought]_V_ [that **[[Ben]**_**NP**_ **[left]**_**VP**_**]**_**S**_]]_VP_]_S_.
3. [[Clara]_NP_ [[said]_V_ [that **[[Anna]**_**NP**_ **[[thought]**_**V**_ **[that** ***[[Ben]***_***NP***_ ***[left]***_***VP***_***]***_***S***_***]*]**_**VP**_**]**_**S**_]]_VP_]_S_.

This process could extend indefinitely, although in practice it is limited by performance constraints imposed by working memory, attention, and other factors, which severely impact understanding after three/four levels of embedding (Christiansen & Chater, 1999, 2016; Cowan, 2010; Lobina, 2011; Miller & Chomsky, 1963). Interestingly, deeper hierarchies can emerge in written texts where memory constraints are less pronounced (Karlsson, 2007).

While the examples above illustrate how recursive hierarchical embedding (RHE) generates complex linguistic structures, the question remains: *what cognitive computations underlie this capacity?* In linguistic theory, particularly within the Minimalist Program, recursion is attributed to a fundamental combinatorial operation called Merge (Berwick & Chomsky, 2016; Chomsky, 2007). Merge applies to two objects—such as lexical items or phrases—and forms a new syntactic unit. What makes Merge recursive is that its output can serve as input again, allowing for unbounded hierarchical growth. Thus, from a small inventory of primitives and rules, Merge yields structures of potentially infinite depth.

Importantly, Merge is not a monolithic process but involves at least two subcomponents (Berwick et al., 2013; Boeckx, 2009, 2012; Hornstein & Pietroski, 2009; Zaccarella et al., 2017). The first is concatenation, the act of combining two objects into a single set or ordered structure X + Y → {X, Y}. The second is labelling, which determines which element serves as the “head” of the new unit and thereby establishes the hierarchical asymmetry X + Y → {X’{X, Y}}, as in the example of [[student]_NP_ committee]_NP_, in which the head is ‘committee’. This distinction is critical: concatenation alone could generate a flat set of elements, but without a process that assigns headedness, there would be no hierarchical embedding. In this sense, recursion arises precisely from the interaction of concatenation and labelling, enabling asymmetric and unbounded embedding. Importantly, within this framework, both the inputs and outputs of Merge are syntactic objects (Chomsky, 2007, 2013), ensuring that the hierarchy can be read by a linguistic processing system and extended further: {X’{X, Y}} + Z → {Z’ {X’{X, Y}}}.

This computational analysis has empirical implications. If recursion depends on the interaction of these subcomponents, then the central question becomes whether concatenation and labelling are unique to language or whether *analogous* processes can be found across cognitive domains such as vision, music, or action planning (Fadiga et al., 2009; Fitch & Martins, 2014; Jackendoff & Lerdahl, 2006; Jackendoff & Pinker, 2005; Martins et al., 2015; Pulvermüller, 2014). One possibility is that non-linguistic recursion requires translating visual or musical primitives into canonical forms as syntactic objects, allowing them to be processed through the language domain-specific architecture of Merge (Fitch et al., 2005; Hauser et al., 2002). An alternative is that multiple cognitive systems each implement their own analogues of concatenation and labelling, yielding a multiply domain-specific architecture (Fedorenko & Shain, 2021). Finally, a more radical view is that the basic computations achieved by what we call concatenation and labelling in language reflect domain-general computations, scaffolded by shared cognitive and neural mechanisms and available across symbolic domains (Christiansen & Chater, 1999; Dehaene et al., 2015, 2022).

Theoretical and empirical advances over the past decade have enabled the study of recursive hierarchical embedding (RHE) beyond language, in domains such as vision, music, and action (Martins, 2012; Martins et al., 2016, 2017; Martins, Bianco, Martins et al., 2019a, b, 2025; Martins, 2024). A key step has been distinguishing RHE from iterative hierarchical embedding (IHE), since both can produce identical nested hierarchies relying on different generative principles. For example, the recursive rule A → A [A A A] could be used to generate the 3-branching hierarchies:

1. A
2. A [A A A]
3. A[A [A A A] A [A A A] A [A A A]]

An iterative rule can generate an identical 3-branching structure by adding elements within fixed hierarchical levels without generating new ones. For example, applying the rule A [B [C]] → A[B [C C]], we could obtain:

1. A [B [C] [B [C] [B [C]],
2. A [B [CC] [B [CC] [B [CC]]
3. A [B [CCC] [B [CCC] [B [CCC]]

**Fig. 1:**
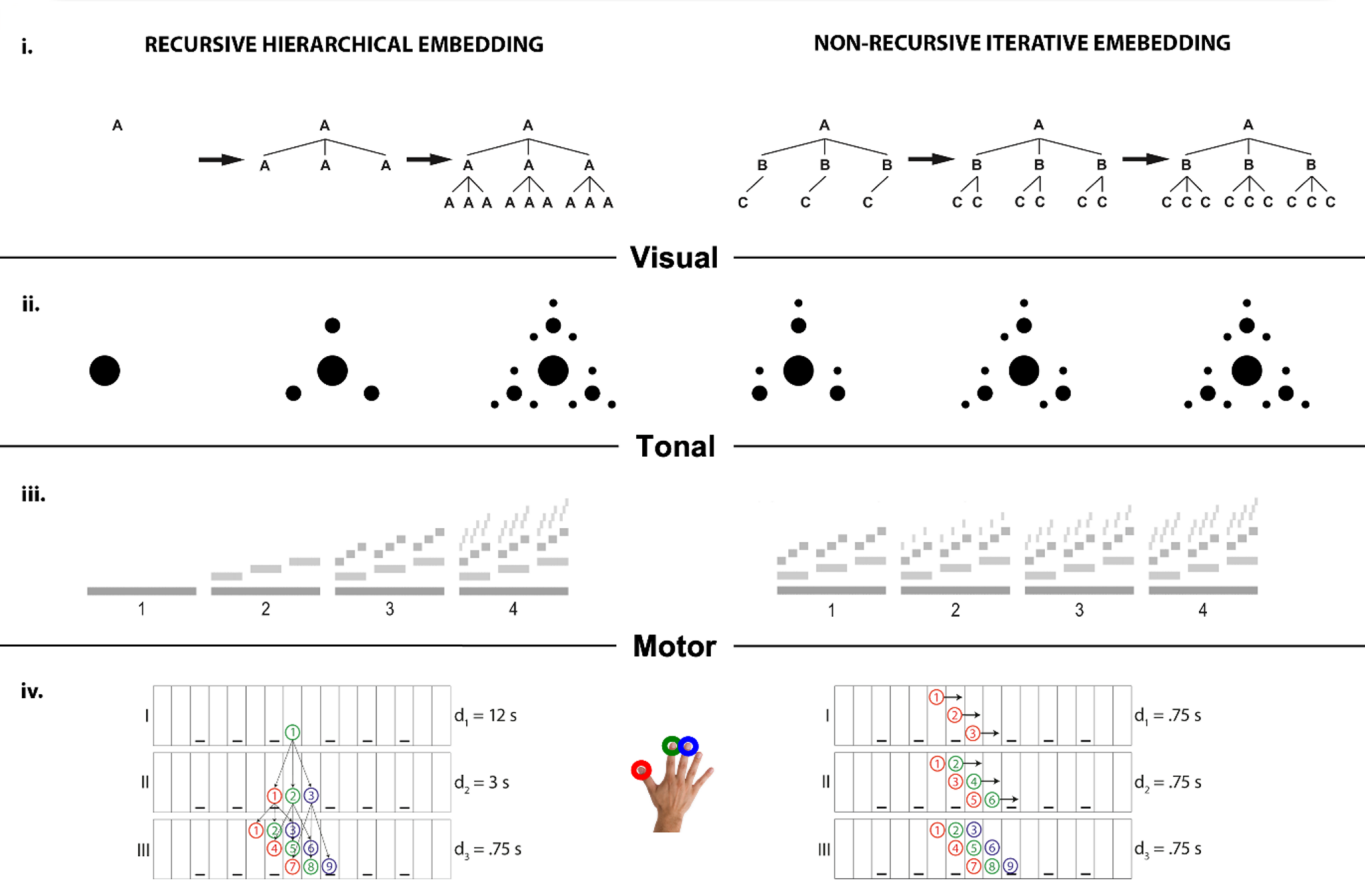
General distinction between recursive hierarchical embedding and non-recursive iteration across domains. Abstract representation of the generative processes (**i**) and its application in the visual (**ii**), tonal (**iii**), and motor (**iv**) domains. Regardless of the generative process, the final structure is perceptually and motorically similar between recursive and iterative conditions, controlling for lower-level features

With this framework, we can target the mental representations involved in representing processes that extend hierarchical levels beyond the given, while maintaining the properties of the stimuli constant. The crucial difference is that IHE constitutes a serial algebraic process that generates (potentially infinite) sets without headedness (as in N_(i)_ = N_(i−1)_ + 1 or concatenation), whereas RHE requires the assignment of hierarchical headedness (as in embedding or labelling). Crucially, the same logic applies across domains (Fig. 1), enabling direct comparisons between visual, tonal, and motor recursion while controlling for low-level perceptual or motor features. 

Following this framework, a research program was conducted across domains, revealing that humans can represent recursion in vision, music, and motor sequencing (Martins et al., 2014b, 2015, 2016, 2017; Martins, Bianco, Martins et al., 2019a, b; Martins, Krause, Martins et al., 2019a, b, 2020, 2025; Martins, 2024). The mapping of the similarities and differences across domains revealed a mixed picture.

Behavioral research has shown that the capacity to acquire RHE (controlling for IHE) across vision, music, and motor sequencing loaded onto a single latent factor (Martins et al., 2017). Moreover, RHE in vision (vs. IHE) correlated with the ability to parse two-level center-embedded sentences in language (Martins, Krause, Martins et al., 2019a, b), suggesting that participants who could extend hierarchies in one domain were also more likely to do so in another. A developmental parallel has also been observed: in both motor sequencing and vision, the acquisition of IHE scaffolds the later mastery of RHE, with attempts to learn RHE first often leading to poorer outcomes (Martins, Laaha, Martins et al., 2014a, b, 2025). This mimics the development path of language in which flat conjunction (e.g., [second] and [red] ball) is acquired before coordination (e.g., [[second] red] ball) (Roeper, 2011).

Conversely, neuroimaging studies with well-trained participants have suggested segregated patterns of activity, with visual, tonal, and motor imagery brain networks being active in the contrast between RHE and IHE (Martins et al., 2019a, 2020; Martins, Fischmeister, Martins et al., 2014a, b). Because neural processing is known to undergo significant changes with extensive training (Chein & Schneider, 2005; Jeon & Friederici, 2013, 2015), other studies have tested participants without prior training on the RHE vs. IHE. Here, brain networks engaged by visual and logical hierarchical tasks included posterior temporal and inferior frontal regions, which are also implicated in linguistic recursion (Martins et al., 2019b; Scholz et al., 2023).

Together, these behavioral and neuroimaging results point to overlaps in the cognitive resources used to implement RHE in language and vision when the latter is untrained. However, they also point to the availability of other, more domain-specific resources with extensive training.

Importantly, these results are correlational and imprecise, as adjacent brain regions can instantiate similar computations without overlap (Fedorenko & Shain, 2021). Other paradigms are more suited to addressing the necessity of shared resources, such as neuropsychological and interference studies. For example, a study with a non-verbal autistic child with limited language comprehension revealed an intact ability to instantiate visual recursion, suggesting that recursive competence can emerge independently of linguistic syntax (Rosselló et al., 2025). Similarly, visual recursion was not affected by concurrent motor or verbal working memory suppression tasks, again pointing to independence across domains (Martins et al., 2015). However, these interference studies have not precisely targeted the computations central to recursion in language, such as the algebraic combination of symbolic elements (of which concatenation is an example), and engagement with the lexical processing system (necessary for labelling). Thus, they cannot be considered definitive tests. It also remains possible that recursion is initially implemented by domain-general resources, while alternative domain-specific implementations may become available when these resources are disrupted (Martins, 2024). This motivates the need for more specific manipulations that may interfere with the hypothesized core operations underlying recursive embedding in language.

### The current study

To address these gaps, we introduced an interference paradigm designed to load cognitive resources that are theoretically adjacent to, but not identical with, the subcomponents of recursive hierarchical embedding (RHE) posited in linguistic theory. Our goal was not to reproduce the operations of labelling or set-Merge, but rather to probe algebraic operations and lexical retrieval systems that many accounts propose as necessary inputs to recursive combinatorial processing.

In the first secondary task, participants performed serial arithmetic (N_(i)_ = N_(i−1)_ – 3), which requires maintaining and updating values across successive operations. Serial arithmetic does not instantiate set-Merge, but it taxes a binary recursive algebraic workspace argued to overlap with the combinatorial mechanisms supporting linguistic recursion (Berwick & Chomsky, 2016; Fitch et al., 2005; Hauser et al., 2002). If hierarchical embedding across domains draws on partially shared symbolic generative resources, then loading this workspace should impair visual recursion. Because serial arithmetic produces a sequence of numbers without a head, it may interfere with both RHE and IHE.

In the second secondary task, participants engage in lexical retrieval, generating tokens of a prompted category (e.g., ‘fruit’ ◊ {*apple*,* banana*,* peach*}). Lexical retrieval is not equivalent to syntactic labelling, but within the Minimalist Program, labelling presupposes access to the lexical interface system that encodes categorial and feature information. If, as some accounts suggest, visual primitives must be internally recoded into lexicalized representations before being processed by language-preferential combinatorial mechanisms to generate headings, then taxing the lexical–conceptual workspace should impede hierarchical processing (Nedergaard et al., 2023). It is important to note that while both RHE and lexical retrieval tax verbal working memory and serial verbal production, interfering with these resources alone did not disrupt visual recursion in a previous study (Martins et al., 2015). Because heading is only necessary to generate hierarchical levels in RHE but not in IHE, we expect lexical retrieval to interfere more strongly with RHE than with IHE.

As controls, we include a no-interference condition and a visuo-spatial (matching-to-sample) interference task, the latter taxing attentional and perceptual load but not directly targeting serial, algebraic, or linguistic resources. By embedding these interference manipulations into tasks that test visual recursion and visual iteration, we can compare their effects on structures that differ in generative principles but are matched in perceptual demands.

This design allows us to adjudicate between competing computational architectures of recursion:

1. Language-specific architecture – Visual recursion requires primitives to be recoded as lexical objects so the linguistic combinatorial system can process them (Fitch et al., 2005; Hauser et al., 2002).
2. Multiply domain-specific architecture – Each domain implements its own analogues of recursion and hierarchical embedding, yielding parallel but distinct systems (Fedorenko & Shain, 2021).
3. Domain-general architecture – Recursive hierarchical embedding is implemented by a single recursive algebraic system, such that symbolic inputs from different domains (e.g., visual, linguistic, numerical) compete for overlapping computational resources (Dehaene et al., 2015, 2022).

By selectively loading lexical and algebraic workspaces and contrasting recursion with iteration, our paradigm provides a principled test of whether visual recursion depends on language-domain-specific, visual-domain-specific, or domain-general mechanisms.

Based on the computational architectures outlined above, we tested the following hypotheses:

#### Hypothesis 1

If recursion is language-specific, then lexical retrieval interference should disrupt performance on the recursive tasks more than on iterative tasks. Serial arithmetic interference may also cause disruption insofar as it engages recursive algebraic operations within the language system.

#### Hypothesis 2

If recursion is multiply domain-specific, then neither lexical retrieval nor serial arithmetic interference should differentially disrupt visual recursion. Recursive and iterative tasks should remain resilient to language-related interference. However, visual interference should disrupt both, reflecting reliance on domain-specific visuospatial resources.

#### Hypothesis 3

If recursion is domain-general but not primarily linguistic, then serial arithmetic interference should impair this recursive algebraic system and disrupt visual recursion more than iteration, whereas lexical retrieval interference should not.

## Methods

Two experiments employed a within-subjects dual-task design. Participants completed one of two primary visual discrimination tasks—a Visual Recursion Task (REC; Experiment 1) or an Embedded Iteration Task (ITE; Experiment 2)—while simultaneously performing one of four interference conditions: no interference (NONE), lexical retrieval (LEX), serial arithmetic (MATH), or visual delayed match-to-sample (VIS). The order of interference conditions was fully counterbalanced across participants.

### Participants

Ninety-six participants were tested across two experiments. Experiment 1 (REC) tested 48 participants (30 females; age range = 21–40 years, *M* = 28.3, *SD* = 5.84). Experiment 2 (ITE) tested another 48 participants (33 females; age range = 19–40 years, *M* = 25.8, *SD* = 5.22). Participants were recruited from a volunteer database maintained by the Berlin School of Mind and Brain at the Humboldt-Universität zu Berlin, Germany. All were native German speakers and reported written and spoken English comprehension. Verbal instructions were delivered in English; written instructions for practice and test trials were provided in German. Participants provided written consent and were compensated 9 euros for participation.

### Stimuli

#### Primary tasks

Each participant completed one of two binary forced-choice visual discrimination tasks, depending on the experiment: the Visual Recursion Task (REC) in Experiment 1 or the Embedded Iteration Task (ITE) in Experiment 2. Trial format and timing were identical across tasks.

##### Visual recursion task (REC)

**Fig. 2:**
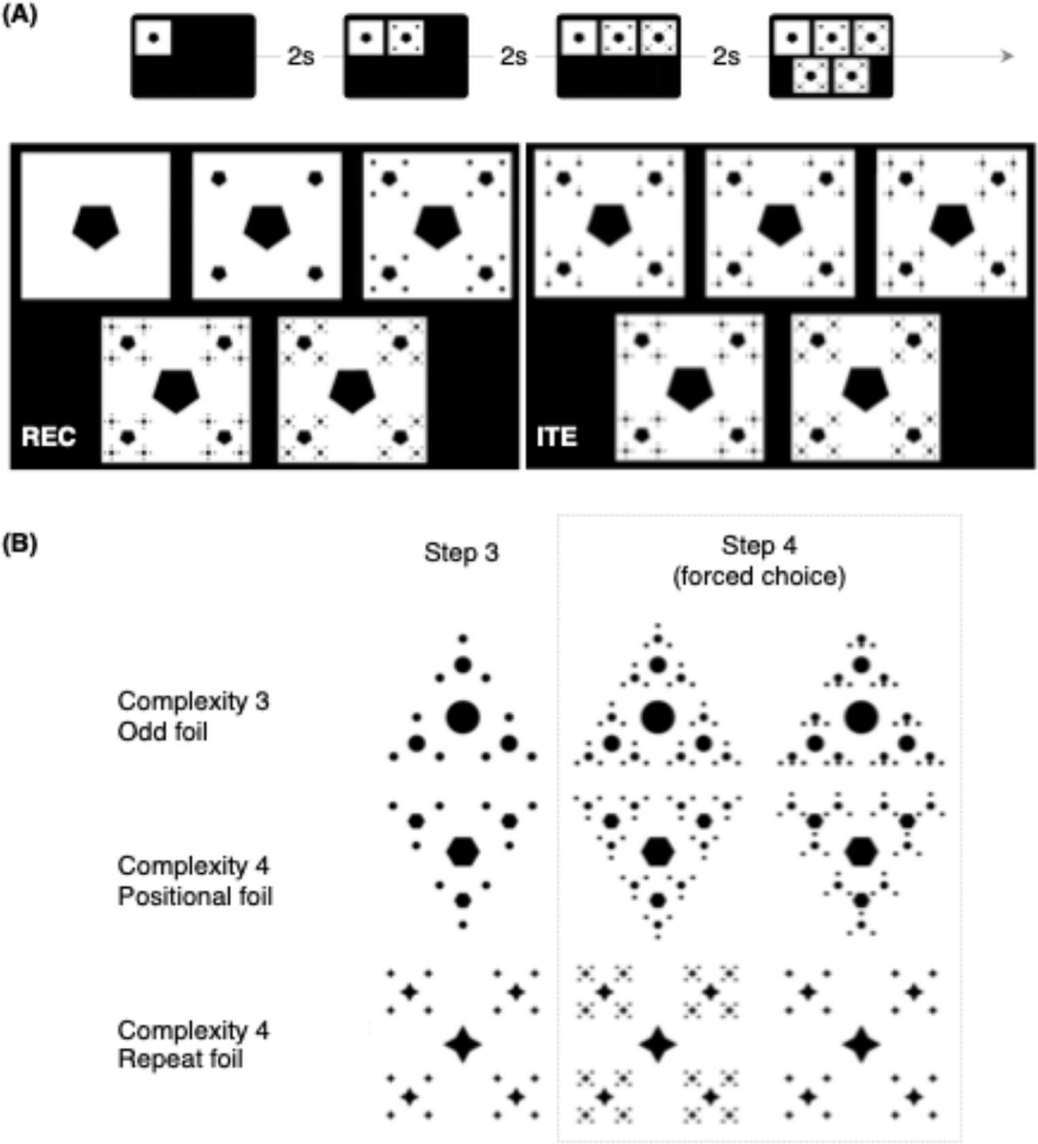
**Task and stimuli examples **(**A**) both the visual recursion task (REC) and the visual iteration task (ITE) presented three images on the upper row, sequentially, in three steps. Then two images were presented in the bottom row, one correct and one foil. (**B**) Trials varied across dimensions of (1) visual complexity (3 and 4), alluding to the number of constituents per root node, and (2) the kind of foils. In odd foils, one constituent per root was misplaced. In positional foils, all constituents around the root were symmetrically rotated. Finally, repeat foils were identical to the image presented in step 3

In the REC task (Fig. 2A), participants viewed a sequence of three images (steps 1–3) depicting the recursive generation of a visual fractal, with each new image presented after a 2-second interval. They were then asked to choose, from two alternatives, the correct continuation of the sequence (step 4). One image followed the recursive embedding rule, while the other was a foil. Participants had 4 s to respond before the trial timeout. In total, each trial lasted 10 s.

Stimuli varied along two orthogonal factors: visual complexity (three or four elements per hierarchical level) and foil type (Fig. 2B). Foils consisted of (a) odd constituent errors (two elements misplaced within the hierarchy), (b) positional errors (all elements internally consistent but inconsistent with the preceding levels), or (c) repetition errors (no additional step after the third iteration). Crossing these factors yielded six stimulus categories, with eight trials per category, for a total of 48 trials.

##### Embedded iteration task (ITE)

The ITE was structurally identical to REC in terms of format and timing, but it tested sensitivity to iterative rather than recursive embedding. Foil types were matched to REC, and visual complexity was balanced across tasks. To equate perceptual demands, half of the correct ITE images matched fractals generated by REC.

REC and ITE have been extensively validated (Martins et al., 2016) and shown to prevent reliance on simple visual heuristics. Previous work further demonstrated that low-level Gestalt cues are insufficient (Martins et al., 2015) and that REC performance correlates with hierarchical reasoning in music, action, language, and logic once ITE effects are controlled (Martins et al., 2017; Martins, Krause, Martins et al., 2019a, b; Scholz et al., 2023).

#### Interference tasks

**Fig. 3:**
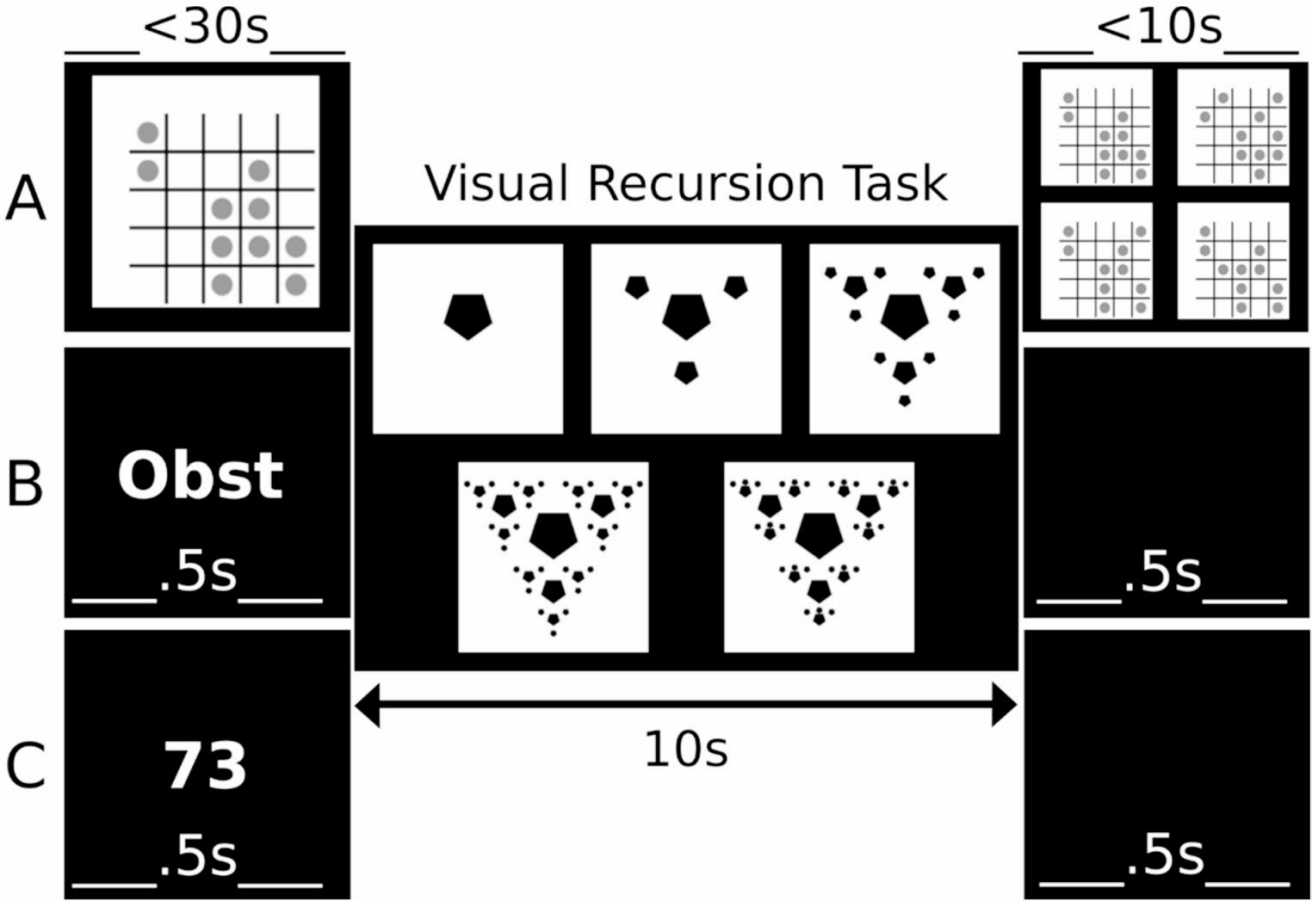
**Interference tasks during both the Visual Recursion Task (REC) and the Visual Iteration Task (ITE). **(**A**)** Visual interference (VIS)**: A checkerboard matrix was presented for up to 30 s before the trial. After making their primary task choice (or timeout), participants had 10 s to identify the original matrix from four options. (**B**)** Lexical retrieval interference (LEX)**: A category label (e.g., *“fruit (Obst in German)”*) appeared for 500 ms. Participants then generated exemplars aloud while completing the REC trial. After responding, a 500-ms blank screen appeared, followed by the subsequent trial. (**C**)** Arithmetic interference (MATH)**: A number (ranging between 35 and 100) appeared for 500 ms. Participants performed successive subtractions aloud (N_(i+1)_ = N_(i)_ – 3) while completing the primary task trial. After responding, a 500 ms inter-trial interval followed

To probe the contribution of linguistic and domain-general resources to visual recursion and iteration, participants performed the primary tasks under four interference conditions (Fig. 3). One baseline condition without interference, and three conditions with the following secondary tasks:

##### Visual delayed match-to-sample (VIS)

Participants memorized a checkerboard pattern presented before each trial (with self-paced continuation, for a maximum of 30 s). Then they identified it among four alternatives (within 10 s) after completing the primary task. This task controlled for visuo-spatial working memory load, without targeting lexical or algebraic resources. The delayed matching-to-sample (DMTS) paradigm is widely used to probe short-term visual memory, recruiting systems involved in visual encoding, storage, and executive control (Daniel et al., 2016). Accuracy in the secondary VIS task was on average M = 0.73 (SD = .17), which is within the range reported in the previous literature (Campos-Magdaleno et al., 2021; Roque et al., 2011).

##### Lexical retrieval (LEX)

A category label (e.g., “vegetables”) was displayed for 500 ms, after which participants generated aloud as many category exemplars as possible while completing the primary task. This manipulation taxed lexical retrieval and semantic search, processes known to support the covert labelling of visual and conceptual material. Such labelling is thought to scaffold both categorization and memory by recoding perceptual inputs into linguistic objects, thereby facilitating discrimination and rehearsal (see Nedergaard et al., 2023, for a review). Verbal interference tasks of this kind are well established to disrupt tasks where labels provide an advantage (e.g., color and object categorization, rule-based category learning), while exerting less consistent effects on tasks that rely on fine visual detail or abstract structural reasoning (Nedergaard et al., 2023). Thus, the LEX condition specifically targeted the verbal workspace implicated in naming and symbolic recoding, processes often hypothesized to be integral to linguistic recursion.

##### Serial arithmetic (MATH)

A random number (35–100) was presented for 500 ms, and participants performed aloud successive subtractions (N_(i)_ = N_(i−1)_ – 3) during the primary task. This task recruits cognitive systems involved in stepwise updating and procedural calculation, overlapping with verbal–phonological processes also engaged during language tasks (Istomina & Arsalidou, 2024; Pollack & Ashby, 2018). It was expected to interfere with hierarchy building by taxing serial updating processes that manipulate symbolic objects.

### Procedure

Before the main experiment, participants received a tutorial and completed six practice trials for each secondary task separately (i.e., not in dual-task format). For the MATH and LEX practices, different numbers and category words were used than in the main experiment to avoid learning effects. For the primary task, they were neither instructed on the concepts of recursion nor iteration beforehand, nor given trial feedback.

During the experiment, each participant completed four 12-trial blocks, one for each interference condition (NONE, VIS, LEX, MATH), while performing the primary task (REC or ITE). The order of interference blocks was fully counterbalanced across participants using a Latin-square design, yielding 24 possible sequences, each completed by exactly two participants (48 per experiment). In total, 96 participants completed the study, producing 4,608 trials.

At the beginning of each interference block, participants were presented with explicit on-screen instructions indicating which secondary task they were required to perform during the upcoming 12 trials. Participants were told that they must perform both the primary and secondary tasks to the best of their ability, and that their responses in both tasks would be recorded and used for analysis.

Within each trial, a secondary-task cue appeared before the primary sequence—a checkerboard matrix (VIS), a category label (LEX), or a number to update (MATH). This pre-trial stimulus served both as a cue and as the content that the participant needed to maintain or produce, clearly signalling that the secondary task was active on that trial. Participants then completed the primary visual discrimination task while concurrently performing the secondary task specified for that block.

Primary-task responses were made via touchscreen within a 4-s response window. Secondary-task responses were produced aloud, recorded as audio files, and continuously monitored by the experimenter to ensure compliance. VIS responses were recorded using the touchscreen at the end of the trial. Short breaks were provided between the 12-trial blocks, and the full session lasted approximately 50 min.

After the dual-task experiment, each participant completed a battery of neuropsychological tests assessing spatial working memory (the backward version of the Corsi block tapping task), verbal working memory (the backward digit span task), and hierarchical planning (the Tower of Hanoi). All tasks were administered with the freely available PEBL software (Mueller & Piper, 2014).

### Analysis

All scripts and datasets are available at https://osf.io/6rp39/files/osfstorage. Analyses of accuracy (proportion correct) and reaction times (RTs; correct trials only) were conducted in R (version 4.4.1) using the *lme4* package (Bates et al., 2014).

Accuracy data were modeled with a binomial logistic mixed-effects regression (*glmer*), and RTs were modeled with linear mixed-effects regression after log-transformation to correct for skew (*lmer*). Planned contrasts tested (a) whether VIS, LEX, and MATH interfered with the primary tasks (vs. NONE) and whether (b) interference conditions differentially affected REC vs. ITE. Random intercepts for participants were included to account for repeated measures.

*P*-values for fixed effects were obtained using *lmerTest*, which applies Satterthwaite’s approximation for denominator degrees of freedom (Kuznetsova et al., 2017). Pairwise contrasts were conducted using the *emmeans* package (Russell, 2018), with Holm–Bonferroni correction for multiple comparisons. Model diagnostics were performed with the *performance* package (Lüdecke et al., 2021). The R Markdown (and editable script) can be inspected on the Open Science Framework https://osf.io/zbrdv.

We also performed an exploratory analysis of foil effects (Repeat, Odd, Positional), which is reported in the supplementary materials (Supplementary Table S3 and Supplementary Figures S1 and S2).

Because the interference effects suggested a clear speed–accuracy trade-off—most notably, LEX and MATH reduced accuracy in both tasks but sped responses in REC—we conducted exploratory analyses using a drift–diffusion model (DDM) (Ratcliff & McKoon, 2008). In such situations, accuracy and RT cannot be interpreted independently, as changes in either measure may arise from different latent components of the decision process. DDM provides a principled framework for decomposing these components (Ratcliff et al., 2016), and hierarchical implementations (HDDM) are particularly well suited for designs with modest trial counts or multiple experimental conditions (Wiecki et al., 2013).

The model jointly estimated three psychologically interpretable parameters: drift rate (v; efficiency of evidence accumulation), boundary separation (a; response caution or decision threshold), and non-decision time (t; perceptual and motor latencies unrelated to the decision). Each parameter was allowed to vary as a function of Task (REC vs. ITE), Condition (NONE, VIS, LEX, MATH), and their interaction, with subject-specific random intercepts and random slopes for each interference condition. Weakly informative priors and log–logit link functions were applied to stabilize estimation and ensure parameter identifiability.

We estimated hierarchical DDMs using the Hierarchical Sequential Sampling Model (HSSM) framework (Fengler et al., 2025), implemented in Python 3.10 with PyMC as the sampling backend. Models were estimated with four chains of 2,000 iterations each (including 2,000 warmup samples), yielding 8,000 posterior draws per parameter. Model convergence was assessed using effective sample size (ESS) and Gelman–Rubin statistics (R-hat). Trace plots are shown in supplementary figures S3-S5.

Given the large sample size, frequentist tests would yield trivially significant p-values even for negligible effects. We therefore adopted a Bayesian approach, which allows us to quantify the degree of evidence for or against an effect. Posterior probabilities (*P* > 0) are reported to indicate the proportion of posterior draws above zero for each effect, corresponding to the probability that the parameter is positive. Following conventional interpretations (Kruschke, 2021; Wagenmakers et al., 2018), values around 0.95 or higher provide strong evidence for a positive effect, values around 0.05 or lower provide strong evidence for a negative effect, and values near 0.5 indicate little to no evidence in either direction.

The complete analysis pipeline, including the Python notebook implementing the HSSM models, is available on the Open Science Framework (https://osf.io/hnu53).

To explore the relationship between task performance and cognitive covariates, we first standardized the behavioral variables. Accuracy and response time (RT) measures from each task condition (None, Visual, Lexical, Math) were z-scored within task. In contrast, cognitive covariates (Corsi block span, Digit Span, Tower of Hanoi) were z-scored across the entire sample. We then fitted a series of linear models predicting accuracy in baseline (NONE) and interference conditions (VIS, LEX, MATH) from the cognitive covariates, task (REC vs. ITE), RT, and their interactions, with specific focus on the task × covariate interaction terms. Outliers were identified and removed based on standardized residuals, and Box–Cox transformations were applied where appropriate (https://osf.io/tq5pa).

## Results

### Accuracy and RT analysis

Raw accuracy and RT are shown in Table 1.

**Table 1:** **Accuracy and RT means across conditions**. *M*: Mean; *SD*: standard Deviation; REC: Recursion; ITE: Iteration; VIS: visual interference; LEX: lexical retrieval; MATH: serial arithmetic; NONE: no interference. Descriptive data are computed across 12 trials per condition and 48 participants per task (12 × 48 = 576). Inferential statistics are based on mixed-effects models that account for trials nested within participants. For a retrospective power analysis, see supplementary table S6 and figures S7-S8

Task	Condition	Trials	Accuracy (M±SD)	RT (M±SD)
ITE	NONE	576 (12 × 48)	0.87±0.33	1.97±0.72
	VIS	576 (12 × 48)	0.83±0.38	2.28±0.80
	LEX	576 (12 × 48)	0.70±0.45	2.25±0.75
	MATH	576 (12 × 48)	0.70±0.46	2.27±0.78
REC	NONE	576 (12 × 48)	0.83±0.38	2.06±0.74
	VIS	576 (12 × 48)	0.85±0.36	2.18±0.70
	LEX	576 (12 × 48)	0.75±0.43	1.92±0.75
	MATH	576 (12 × 48)	0.78±0.41	1.97±0.71

**Fig. 4:**
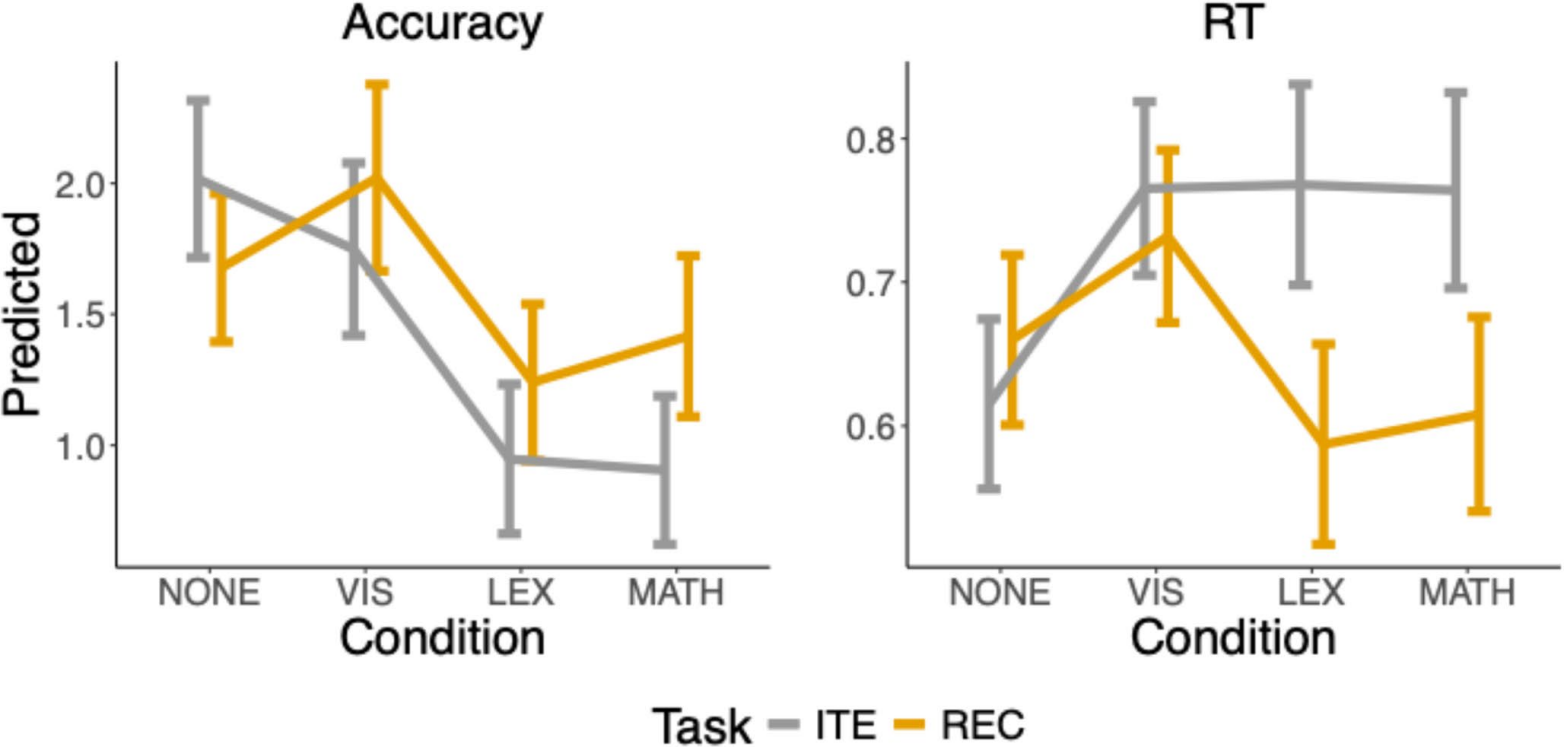
Predicted accuracy (probability) from the mixed-effects models across interference conditions for accuracy and response times (RT). Lines show the differences between the Recursion (REC) and Iteration (ITE) tasks, as well as between interference conditions: visual interference (VIS), lexical retrieval (LEX), serial arithmetic (MATH), and no interference (NONE). Error bars represent 95% confidence intervals

The model results are presented in tables 2; Fig. 4. Overall, we found a significant interaction task ⋅ condition for both accuracy and reaction time. Against our predictions, all interference conditions (visual, lexical, and arithmetic) *decreased* the accuracy and *increased* the RT of iteration (ITE) more than in recursion (REC). Results were broadly identical when including the effects of foil (Supplementary table S3, figures S1 and S2).

**Table 2:** Results of mixed-effects models for accuracy and reaction times (RT). Accuracy was analyzed using logistic regression and reported as odds ratios (OR) with 95% confidence intervals (CI). Reaction times (log-transformed) were analyzed using linear regression and reported as unstandardized coefficients (B) with 95% CIs. Task (reference = ITE) and condition (reference = NONE) were entered as fixed effects, with subject-specific random intercepts and slopes for condition. Significant interaction effects indicate that the impact of interference conditions differed between the recursion (REC) and iteration (ITE) tasks. VIS: visual interference, LEX: lexical retrieval, MATH: serial arithmetic; NONE: no interference

Predictors	Acc			RT		
	OR	CI	*p*	B	CI	*p*
(Intercept)	7.76	5.67–10.62	**< 0.001**	0.61	0.56–0.66	**< 0.001**
Task [REC-ITE]	0.72	0.47–1.10	0.132	0.04	−0.03–0.12	0.228
Condition [VIS-NONE]	0.71	0.51–0.99	**0.041**	0.15	0.11–0.19	**< 0.001**
Condition [LEX-NONE]	0.32	0.24–0.44	**< 0.001**	0.15	0.11–0.19	**< 0.001**
Condition [MATH-NONE]	0.31	0.23–0.43	**< 0.001**	0.14	0.10–0.19	**< 0.001**
[REC-ITE] × [VIS-NONE]	1.77	1.11–2.82	**0.016**	−0.08	−0.14 – −0.02	**0.008**
[REC-ITE] × [LEX-NONE]	1.86	1.21–2.86	**0.005**	−0.23	−0.29 – −0.17	**< 0.001**
[REC-ITE] × [MATH-NONE]	2.28	1.48–3.52	**< 0.001**	−0.20	−0.26 – −0.14	**< 0.001**

#### Accuracy

Pairwise comparisons (Supplementary Table 1; Fig. 4) show that visual interference (VIS) did not reliably reduce accuracy in either task. For iteration (ITE), accuracy was slightly lower under VIS compared to NONE, but the effect did not reach statistical significance (*B* = 0.35, *SE* = 0.17, *p* =.081). In recursion (REC), accuracy even tended to be higher under VIS, though again non-significant (*B* = − 0.22, *SE* = 0.17, *p* =.357).

By contrast, lexical retrieval (LEX) and arithmetic (MATH) robustly impaired accuracy in ITE relative to NONE (LEX: *B* = 1.13, SE = 0.16, *p* <.001; MATH: *B* = 1.16, *SE* = 0.16, *p* <.001). In REC, LEX also reduced accuracy (*B* = 0.51, *SE* = 0.15, *p* =.004), whereas the negative effect of MATH was weaker (*B* = 0.34, *SE* = 0.15, *p* =.089).

Comparisons between interference types further revealed that both LEX and MATH yielded significantly lower accuracy than VIS in both REC and ITE (all ps ≤ 0.002). In contrast, accuracy did not differ between LEX and MATH (both *p*s ≥ 0.357).

Overall, lexical retrieval and arithmetic interference reliably reduced accuracy in both tasks, with the strongest decrements observed in the iteration task. By contrast, visual interference had little or no impact on accuracy.

#### RT

Pairwise comparisons (Supplementary Table 2; Fig. 4) show that VIS significantly increased RT in both REC (*B* = − 0.07, *SE* = 0.02, *p* =.003) and ITE (*B* = − 0.15, *SE* = 0.02, *p* <.001).

In ITE, both LEX (*B* = − 0.15, *SE* = 0.02, *p* <.001) and MATH (*B* = − 0.14, *SE* = 0.02, *p* <.001) also produced slower responses, with no reliable differences between VIS, LEX, and MATH (all *p*s = 1.00).

By contrast, in REC, LEX (*B* = 0.08, *SE* = 0.02, *p* <.001) and MATH (*B* = 0.05, *SE* = 0.02, *p* =.020) were associated with slightly *faster* responses than NONE, opposite to the pattern in ITE. This divergence is highlighted in the contrasts with VIS: RTs were significantly shorter for both LEX (*B* = 0.15, *SE* = 0.02, *p* <.001) and MATH (*B* = 0.12, *SE* = 0.02, *p* <.001) in REC, whereas they were virtually identical in ITE (all *p*s = 1.00).

Overall, all interference conditions slowed responses in iteration, but in recursion, lexical retrieval and arithmetic slightly sped up responses compared to baseline, while visual interference produced slower responses. This suggests that recursion and iteration employ distinct processing strategies, with recursion exhibiting selective resistance to time costs resulting from lexical- and arithmetic-related interference.

#### Drift-diffusion model

This interesting pattern of results suggests that secondary tasks might have differently impacted the cognitive strategy used in recursion and iteration. To explore these dynamics, we ran an exploratory drift diffusion model, which combines accuracy and RT dynamics into a cognitive model of information accumulation and decision processes. In particular, we model the drift rate (information accumulation speed), boundary separation (the amount of evidence needed to make a decision), and non-decision time (the time required for perceptual and motor processes).

**Fig. 5:**
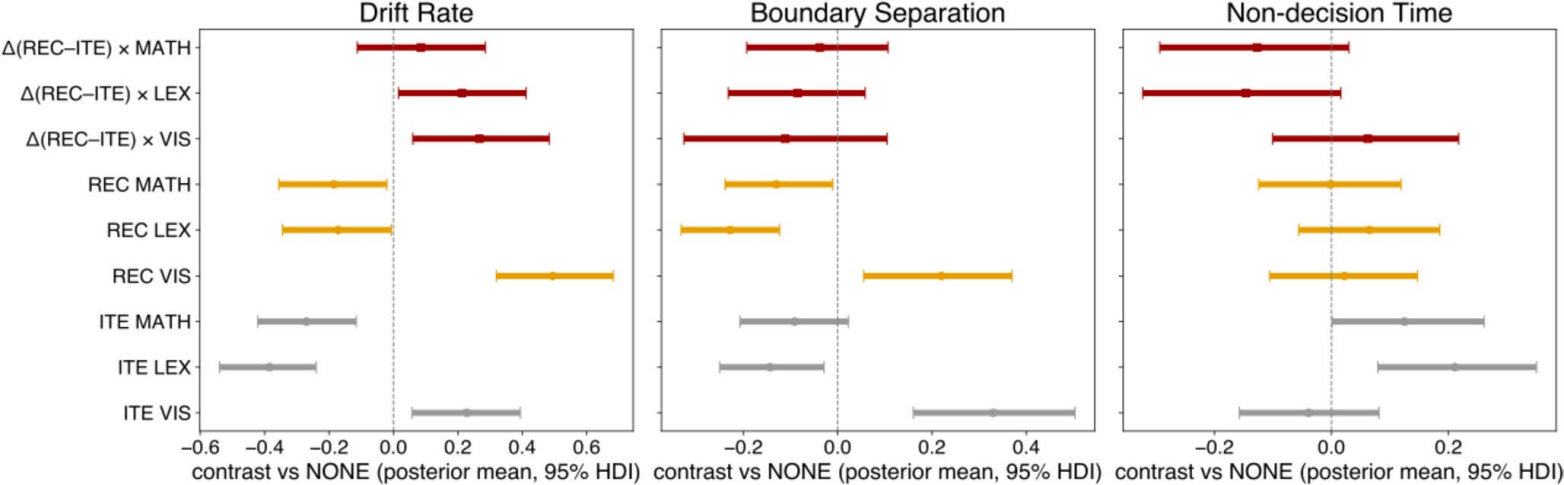
Posterior estimates from the drift diffusion model (DDM). Forest plots display contrasts of drift rate (*v*), boundary separation (*a*), and non-decision time (*t*) between each interference condition (VIS: visual interference; LEX: lexical retrieval; MATH: serial arithmetic) and the baseline (NONE), separately for iteration (ITE) and recursion (REC). Squares indicate posterior means, horizontal bars show 95% highest density intervals (HDI). Orange markers correspond to REC, gray markers to ITE, and brown markers to task × condition interaction effects (ΔREC–ITE). All contrasts are expressed relative to the baseline condition (NONE) within task, or as task differences (REC–ITE) within condition

Model convergence was satisfactory (Min ESS = 860; Max R-hat = 1.004) (Supplementary Figure S3-S5). Posterior estimates for drift rate (*v*), boundary separation (*a*), and non-decision time (*t*) are summarized in Supplementary Table S4 and visualized in Fig. 5.

##### Drift (***v***)

Lexical retrieval (LEX) and arithmetic interference (MATH) consistently reduced the speed of evidence accumulation in both iteration (ITE) and recursion (REC). For ITE, there was very strong evidence for slower drift in LEX (*M* = − 0.39, 95% HDI [–0.54, − 0.24], *P* < 0 = 1.00) and MATH (*M* = − 0.27, 95% HDI [–0.42, − 0.12], *P* < 0 = 1.00). The same pattern held for REC, albeit with smaller effect sizes (LEX: *M* = − 0.17, 95% HDI [–0.34, − 0.01], *P* < 0 =.98; MATH: *M* = − 0.19, 95% HDI [–0.36, − 0.02], *P* < 0 =.98). By contrast, visual interference (VIS) increased drift in both tasks, with very strong evidence in both ITE (*M* = 0.23, 95% HDI [0.06, 0.39], *P* > 0 =.99) and REC (*M* = 0.50, 95% HDI [0.32, 0.68], *P* > 0 = 1.00).

Importantly, interactions showed that these changes were task-sensitive. The increase in drift under VIS was more pronounced in REC than ITE (*M* = 0.27, 95% HDI [0.06, 0.48], *P* > 0 =.99), while the reductions under LEX were less severe in REC than ITE (*M* = 0.21, 95% HDI [0.02, 0.41], *P* > 0 =.98). For MATH, evidence for a task difference was weaker (*M* = 0.09, 95% HDI [–0.11, 0.29], *P* > 0 =.80).

These results indicate that lexical and arithmetic interference impaired the efficiency of information accumulation, whereas visual interference enhanced it. Moreover, recursion was relatively more resistant to interference-induced reductions in drift and more sensitive to facilitation by visual interference.

##### Boundary (***a***)

A parallel pattern was observed for boundary separation. VIS increased decision thresholds for both ITE (*M* = 0.33, 95% HDI [0.16, 0.50], *P* > 0 = 1.00) and REC (*M* = 0.22, 95% HDI [0.06, 0.37], *P* > 0 = 1.00). By contrast, LEX lowered thresholds in both tasks, with strong evidence in ITE (*M* = − 0.14, 95% HDI [–0.25, − 0.03], *P* < 0 =.99) and very strong evidence in REC (*M* = − 0.23, 95% HDI [–0.33, − 0.12], *P* < 0 = 1.00). MATH showed a reduction in REC (*M* = − 0.13, 95% HDI [–0.24, − 0.01], *P* < 0 =.99) and weak evidence of a reduction in ITE (*M* = − 0.09, 95% HDI [–0.21, 0.02], *P* < 0 =.94).

Task × condition interactions were less clear, with most posterior mass overlapping zero (all *P*(>|0|) < 0.85).

These findings suggest that visual interference promoted more cautious responding (higher thresholds). In contrast, lexical and arithmetic interference encouraged less cautious responding (lower thresholds), consistent with a strategic adjustment in decision criteria under different secondary task demands.

##### Non-decision time (***t***)

Non-decision components revealed a different profile. In ITE, there was strong evidence that LEX (M = 0.21, 95% HDI [0.08, 0.35], *P* > 0 = 1.00) and MATH (M = 0.13, 95% HDI [0.00, 0.26], *P* > 0 =.97) prolonged perceptual/motor processing, whereas VIS had little effect (M = − 0.04, HDI spanning zero). In REC, by contrast, all interference effects were close to zero, with wide HDIs.

Critically, one interaction suggested moderate differential task sensitivity: LEX increased non-decision time more strongly in ITE than REC (M = − 0.15, 95% HDI [–0.32, 0.02], *P* < 0 =.95). Other task differences showed weaker evidence.

This pattern indicates that lexical and arithmetic interference selectively increased perceptual or motor latencies in ITE, whereas REC was largely unaffected in these non-decisional processes.

### Exploratory analysis with cognitive assessment

At the end of each experiment, participants performed a short cognitive assessment of spatial working memory (Corsi block tapping), verbal working memory (digit span), and hierarchical planning (Tower of Hanoi). Broadly, accuracy in these tasks predicted increased accuracy in REC/ITE (Supplementary Figure S6). To explore whether these cognitive abilities impacted REC and ITE differently across conditions, we ran linear models controlling for RT (Supplementary Table S5). Verbal working memory correlated more strongly with REC than with ITE. However, this effect was only significant for the no-interference condition (*B* = 0.39, *SE* = 0.18, *p* =.04), while remaining below threshold for MATH (*B* = 0.30, *SE* = 0.18, *p* <.1). There were no other significant differences between tasks (all *p*s> 0.1).

## Discussion

This study tested whether visual recursion (REC) interacts with linguistic, visuospatial, or more general symbolic resources using a dual-task interference paradigm. Participants performed a recursive (REC) or an iterative (ITE) visual discrimination task while concurrently engaging in lexical retrieval (LEX), serial arithmetic (MATH), visual delayed match-to-sample (VIS), or no interference (NONE). Two main empirical patterns emerged. First, LEX and MATH reliably reduced accuracy in both tasks, with larger decrements for ITE than REC. Second, the time costs of interference diverged across tasks: LEX and MATH sped responses in REC but slowed them in ITE. Drift–diffusion modeling (DDM) aligned with these observations: LEX and MATH decreased drift rates (less efficient evidence accumulation) and lowered decision thresholds (less cautious responding) in both tasks, with drift decrements larger for ITE; VIS, by contrast, increased both drift and boundary separation (more efficient but more cautious accumulation). Finally, verbal working memory predicted REC more strongly than ITE at baseline, consistent with linguistic involvement, but this association did not explain ITE’s greater interference sensitivity to LEX and MATH.

Taken together, these results do not fully support any single architecture in our set, but they constrain their plausibility.

Multiply domain-specific recursion (Hypothesis 2) is the least compatible with the data. If visual recursion relied exclusively on visuospatial mechanisms independent from linguistic or algebraic systems, LEX and MATH should have mainly been inert; instead, both impaired performance in REC and ITE. Moreover, VIS—the visuo-spatial control—did not impair accuracy in REC and, in fact, was associated with more cautious accumulation, further weakening a strict visual-only account.

A domain-general symbolic architecture (Hypothesis 3) fares better: the robust impact of MATH on both tasks matches the idea that stepwise manipulation of symbolic items competes for shared computational resources (Dehaene et al., 2015, 2022). However, this account struggles with two aspects of our pattern. First, LEX was at least as disruptive as MATH, despite MATH’s heavier procedural updating demands, suggesting that lexical access is a critical bottleneck (Nedergaard et al., 2023; Pollack & Ashby, 2018). Second, prior work shows that verbal memory interference (7-digit backwards digit span) does not impair visual recursion (Martins et al., 2015), arguing against a generic effect of verbal suppression, memory, and executive control.

A language-preferential architecture (Hypothesis 1) aligns with several observations but requires nuance. LEX taxed the lexical–conceptual workspace, which linguistic theories identify as a prerequisite for assigning labels and establishing asymmetric groupings in syntax, even though lexical retrieval itself does not instantiate the labelling operation (Berwick et al., 2013; Berwick & Chomsky, 2016; Hornstein & Pietroski, 2009). Its substantial interference suggests that, even in vision, organizing elements into structured sets may depend on recoding them as lexical objects before feature-based grouping. Significantly, LEX impaired both REC and ITE, indicating that iteration, although not generating new hierarchical depth, may nonetheless require boundary marking between levels and serial maintenance of groupings (A[B] ◊ A[BB] ◊ A[BBB]). Finally, MATH interference is also compatible with this account, insofar as it loads serial symbolic updating operations that may overlap with those supporting hierarchical combination, without implying that arithmetic instantiates linguistic concatenation itself.

### Why iteration is more vulnerable to interference than recursion

**Fig. 6:**
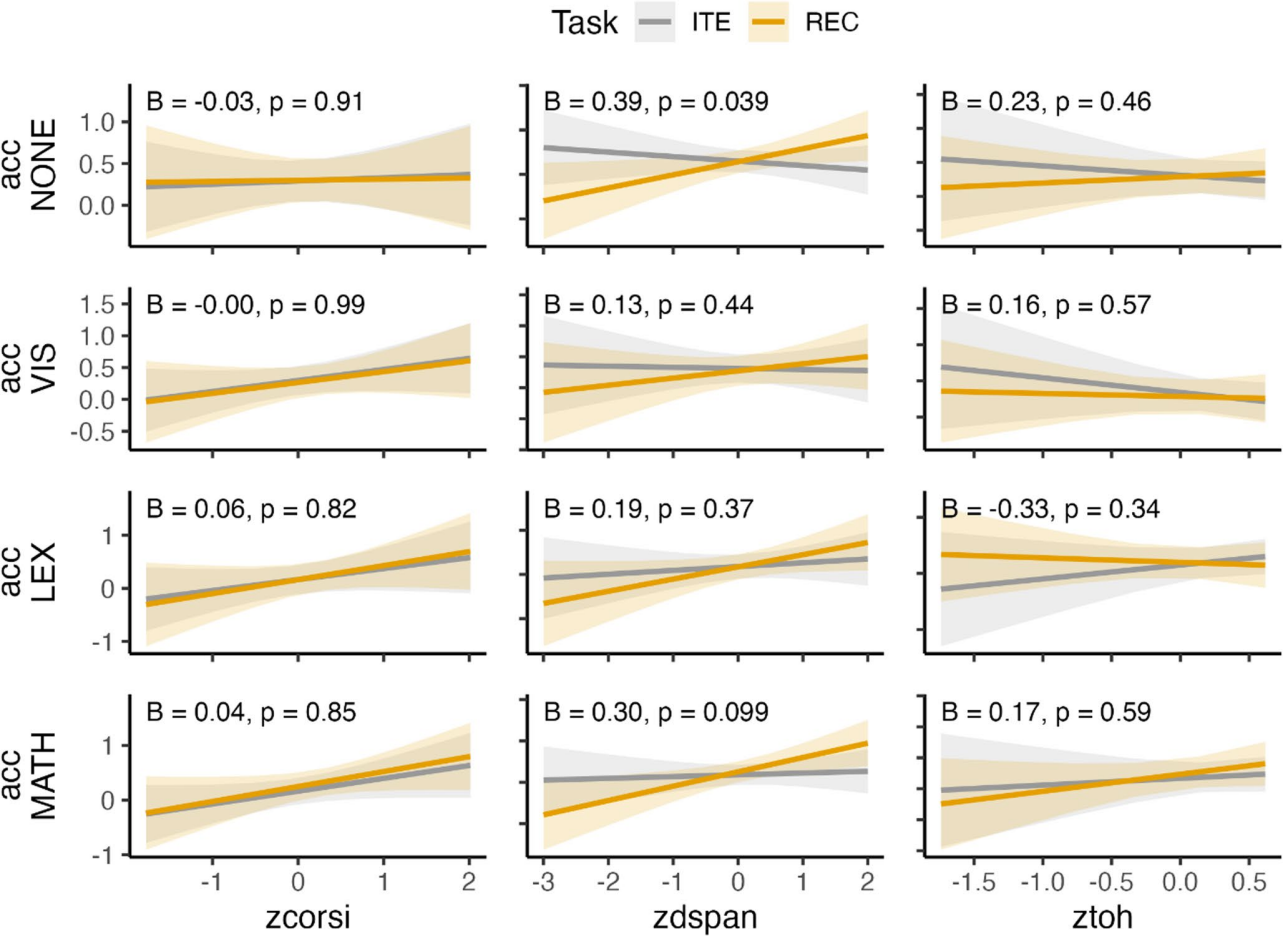
**Linear models predicting accuracy in baseline (NONE) and interference conditions (VIS**,** LEX**,** MATH) from the cognitive covariates (Corsi**,** Digit span**,** Tower of Hanoi)**,** task (REC vs. ITE)**,** RT**,** and their interactions**,** with specific focus on the task × covariate interaction terms (Supplementary Table **S5**).** NONE = no interference; VIS = visual interference; LEX = lexical retrieval; MATH = serial arithmetic; Corsi = Corsi block tapping (spatial working memory); Digit Span (verbal working memory task); ToH = Tower of Hanoi (Hierarchical planning task)

A striking feature of the present results is that iteration (ITE) was more strongly disrupted than recursion (REC) by both lexical retrieval and serial arithmetic. This asymmetry is puzzling, given that REC (vs. ITE): (1) shows stronger associations with verbal working memory at baseline (Fig. 6), (2) correlates more strongly with the ability to parse long-distance dependencies in language (Martins et al., 2019), and (3) engages the language brain network more robustly (Martins et al., 2019). Moreover, REC not only showed better resistance to interference but also faster RTs in LEX and MATH. To explain this unexpected result, we hypothesize that two distinct strategies can support performance in REC, whereas only one appears available for ITE.

In REC, participants can rely on a serial symbolic strategy—likely verbal or algebraically mediated—that incrementally tracks hierarchical depth. This strategy supports higher accuracy and shows strong associations with verbal working memory, consistent with earlier behavioral and neural findings. However, when these symbolic resources are strained (e.g., under LEX or MATH interference), participants may shift to a parallel, visual-configural strategy. This switch is reflected in faster RTs under interference and is compatible with classic global-precedence and configural-processing accounts (Kimchi, 1992, 2014; Navon, 1977). Visual recursion may be buffered by early, short-lived parallel analyses that facilitate rapid decision-making even under symbolic load (Li et al., 2020; Rousselet et al., 2004). The possibility of dual strategies aligns well with Paivio’s dual-coding theory (Paivio, 1969, 1990), which posits that visual and verbal codes can provide alternative representations, and with Chrysikou’s matched-filter framework (Chrysikou et al., 2014), which posits that cognitive control adapts processing modes to task demands.

By contrast, ITE modifies only local structures within a fixed global frame and thus provides no globally distinctive cues that would support configural processing. Each iterative step requires tracking small, distributed changes within the same overall shape. This inherently favors a serial, symbolic maintenance strategy, leaving participants more vulnerable to interference from tasks that also demand symbolic updating and controlled retrieval, such as serial subtraction (Istomina & Arsalidou, 2024; Pollack & Ashby, 2018). Because no parallel-configural alternative is available, interference produces both slower responses and larger accuracy costs. In this view, ITE’s greater susceptibility reflects its tighter dependence on symbolic and sequential resources.

This division resonates with neurocognitive evidence (Fischmeister et al., 2017; Martins et al., 2014a): visual recursion preferentially engages the default mode network (DMN), implicated in abstraction, internal model construction, and distributed semantic integration (Margulies et al., 2016), whereas iteration recruits the frontoparietal network (FPN), associated with serial control and externally oriented effortful processing (Duncan, 2010; Fedorenko et al., 2013; Shashidhara et al., 2019; Sigman & Dehaene, 2008).

Developmental and neuropsychological evidence further supports this interpretation. Iteration precedes recursion in acquisition (Martins et al., 2014b; Roeper, 2011), suggesting a scaffolding role for serial, symbolic, and combinatorial processes. Yet recursion can emerge even in the absence of strong linguistic abilities, as shown by a non-verbal autistic child who performed well in visual recursion but had slightly lower scores in iteration (Rosselló et al., 2025). Together, these findings suggest that recursion is preferentially scaffolded by linguistic and algebraic mechanisms, with alternative compensatory pathways available. In contrast, iteration remains more rigidly tied to serial symbolic processing, explaining its greater vulnerability to interference.

### Alternative explanations

One possibility is that the interference effects of LEX and MATH reflect general attentional or executive resource demands, rather than specific disruption of hierarchical processing. Both tasks are widely used in neuropsychological assessment because they strongly recruit sustained attention, working memory updating, and controlled retrieval (Istomina & Arsalidou, 2024; Nedergaard et al., 2023; Pollack & Ashby, 2018; Shao et al., 2014). From this perspective, interference arises because REC and ITE both depend on limited-capacity executive resources, which are depleted when participants must simultaneously generate words or perform serial arithmetic. This domain-general account can readily explain why both recursive and iterative tasks were affected.

However, three features of the data challenge a pure general-load interpretation. First, earlier work shows that secondary verbal and working memory tasks (backward digit span and finger tapping)—that also tax memory and attention—do not impair visual recursion (Martins et al., 2015). The selective sensitivity to lexical retrieval and symbolic updating points toward qualitative rather than purely quantitative competition. Second, LEX was at least as disruptive as MATH, despite MATH’s higher demands on executive control (Istomina & Arsalidou, 2024; Shao et al., 2014), suggesting that interference is not simply proportional to load. Finally, although baseline ITE performance was less correlated with verbal WM than REC, it was more sensitive to LEX and MATH.

The lack of interference from the visual delayed match-to-sample task (VIS) also raises questions. VIS is itself demanding, but it may bias attention toward visual encoding without imposing strong serial-symbolic requirements, leading to facilitation rather than disruption. Evidence from visual working memory supports this view: maintaining a visual representation can enhance perceptual signal strength and improve discrimination (Pan & Zhang, 2020). Thus, VIS may have acted more as a perceptual prime than as a competing executive task.

Finally, a more skeptical view is that neither REC nor ITE genuinely tap hierarchical processing, and that interference effects simply reflect general distraction from complex secondary tasks. Yet this interpretation is inconsistent with converging evidence: controlling for ITE, REC correlates with hierarchical reasoning in language, logic, music, and action (Martins et al., 2017; Martins, Krause, Martins et al., 2019a, b; Scholz et al., 2023), recruits left IFG and pMTG (Martins, Krause, Martins et al., 2019a, b), and follows a developmental trajectory similar to language and distinct from shallow pattern-matching (Martins, Laaha, Martins et al., 2014a, b).

In sum, domain-general load and visual priming mechanisms likely contribute to the observed effects. But the selective impact of lexical retrieval and serial arithmetic suggests that, beyond general attentional depletion, interference specifically targets serial symbolic generative processes, which appear to be the critical bottleneck in visual hierarchy building.

#### Limitations and future directions

Several limitations should be acknowledged. First, the sample size (*N* = 48 per experiment, 12 trials per condition) was modest. Although the observed effects were robust, retrospective power analyses (Table S6, https://osf.io/6rp39/files/djhqt) indicated that most key interaction effects—particularly for LEX and MATH in both accuracy and RT—had moderate to high estimated power (≥ 0.79–0.99). However, the accuracy contrast between VIS and NONE showed lower power (~ 0.68), and non-significant results in this condition should therefore be interpreted with caution. Simulation-based power curves varying trial number and sample size (Figures S7–S8) further showed that increasing trials to 16 per condition or sample size to roughly 75 participants per experiment would bring all of these effects above the conventional 80% power threshold. Although collecting additional data was not feasible for the present study, these analyses provide a useful benchmark for future experiments and for investigations of more subtle effects or individual-difference moderators.

Second, while LEX and MATH were intended to load cognitive resources adjacent to the subcomponents of Merge—namely, lexical access for labelling and symbolic updating for concatenation—they are not direct probes of these computations. Lexical retrieval taxes access to the mental lexicon, but it does not directly instantiate the labelling of syntactic objects. Similarly, serial subtraction requires recursive algebraic updating, but not recursive set formation. Future work could refine this approach by developing secondary tasks that more closely target recursive operations, such as hierarchical category generation (e.g., animal → mammal → dog → husky) or algebraic manipulation tasks involving nested bracketing.

Furthermore, while we assume that ITE did not require the assignment of hierarchical heads, this might not be the case. Theoretically, it is possible that IHE also requires the representation of the head A’ to process the addition of B visual elements nested within the appropriate hierarchical level A’[B] → A’[BB] → A’[BBB]. If this is the case, future research could employ a third control task involving set formation without headings, i.e., [B] → [BB] → [BBB].

Finally, although the visual interference task did not impair performance, its facilitatory effect raises questions about the boundary conditions under which visual working memory competes with or primes visual hierarchical processing. Parametrically varying the timing, complexity, or similarity of visual interference stimuli could help disentangle these mechanisms.

Finally, while our paradigm isolates symbolic interference effects more precisely than earlier studies, it cannot establish whether linguistic-like mechanisms are obligatory for visual recursion or only preferential. Combining interference tasks with neuroimaging, neuropsychological populations, or developmental designs would allow stronger tests of whether visual recursion flexibly recruits symbolic, domain-general, and visuospatial systems depending on task demands.

## Conclusion

This study shows that visual hierarchical processing depends on serial symbolic generative resources, as both lexical retrieval and arithmetic interference impaired performance in recursion (REC) and iteration (ITE). Iteration proved more vulnerable, whereas recursion was more resilient, likely due to compensatory strategies such as parallel or global processing. Visual interference instead facilitated performance, highlighting the specificity of symbolic rather than visuospatial demands. Together, these findings challenge strictly domain-specific or purely domain-general accounts, supporting instead a hybrid architecture in which recursion preferentially engages language-like mechanisms but can flexibly recruit alternative resources when symbolic systems are taxed.

## Supplementary Information

Below is the link to the electronic supplementary material.

Supplementary Material 1 (DOCX 2.68 MB)

## Data Availability

All data and scripts supporting the findings are available on the Open Science Framework (OSF) at https://osf.io/6rp39/files/osfstorage.

## Code availability

All analysis code (R and Python notebooks) is publicly available on OSF at https://osf.io/6rp39/files/osfstorage.
