# Supplementary material for: Recursion beyond language: Lexical and arithmetic interference in visual hierarchical embedding
Source: Psychol Res. 2026 Mar 10;90(2):54. doi: 10.1007/s00426-026-02252-2 (PMC12975858; doi:10.1007/s00426-026-02252-2)
Supplement: Supplementary file 1 — Supplementary Material 1 (DOCX 2.68 MB) [file 426_2026_2252_MOESM1_ESM.docx]

**Recursion Beyond Language: Lexical and Arithmetic Interference in Visual Hierarchical Embedding**

Mauricio J.D. Martins^1^, Daniel Cook^2^, Arno Villringer^3,4,5^

^1^SCAN-Unit, Department of Cognition, Emotion, and Methods in Psychology, Faculty of Psychology, University of Vienna, Austria

^2^ Technology Infrastructure Department, Site Reliability Engineering Group, Major League Baseball, New York, NY

^3^ Berlin School of Mind and Brain, Humboldt Universität zu Berlin, Berlin, Germany

^4^ Max Planck Institute for Human Cognitive and Brain Sciences, Leipzig, Germany

^5^ Clinic for Cognitive Neurology, University Hospital Leipzig, Germany

Corresponding author:

Maurício Martins, SCAN-Unit, Department of Cognition, Emotion, and Methods in Psychology, Liebiggasse 5, 1090 Wien, Austria

[mauricio.martins@univie.ac.at](mailto:universo.enfim@gmail.com%20%5Ch)

|  |  | Accuracy |  |  |  |
| --- | --- | --- | --- | --- | --- |
| contrast | Task | *estimate* | *SE* | *z* | *p* |
| NONE - VIS | ITE | 0.35 | 0.17 | 2.05 | 0.081 |
| NONE - LEX | ITE | 1.13 | 0.16 | 7.12 | **<0.001** |
| NONE - MATH | ITE | 1.16 | 0.16 | 7.36 | **<0.001** |
| VIS - LEX | ITE | 0.78 | 0.15 | 5.28 | **<0.001** |
| VIS - MATH | ITE | 0.81 | 0.15 | 5.53 | **<0.001** |
| LEX - MATH | ITE | 0.04 | 0.13 | 0.27 | 0.789 |
| NONE - VIS | REC | -0.22 | 0.17 | -1.35 | 0.357 |
| NONE - LEX | REC | 0.51 | 0.15 | 3.33 | **0.004** |
| NONE - MATH | REC | 0.34 | 0.15 | 2.17 | 0.089 |
| VIS - LEX | REC | 0.73 | 0.16 | 4.62 | **<0.001** |
| VIS - MATH | REC | 0.56 | 0.16 | 3.49 | **0.002** |
| LEX - MATH | REC | -0.17 | 0.14 | -1.17 | 0.357 |

**Table S1. Pairwise differences for the model Accuracy = condition × task + (1|ID). Primary tasks:** REC: Visual Recursion Task; ITE: Visual Iteration Task; **Secondary tasks:** VIS: Visual interference, LEX: lexical retrieval, MATH: serial arithmetics; NONE: no interference.

|  |  | RT |  |  |  |
| --- | --- | --- | --- | --- | --- |
| contrast | Task | *estimate* | *SE* | *z* | *p* |
| NONE - VIS | ITE | -0.15 | 0.02 | -6.95 | **<0.001** |
| NONE - LEX | ITE | -0.15 | 0.02 | -6.96 | **<0.001** |
| NONE - MATH | ITE | -0.14 | 0.02 | -6.66 | **<0.001** |
| VIS - LEX | ITE | 0.00 | 0.02 | 0.02 | 1.000 |
| VIS - MATH | ITE | 0.01 | 0.02 | 0.26 | 1.000 |
| LEX - MATH | ITE | 0.01 | 0.02 | 0.24 | 1.000 |
| NONE - VIS | REC | -0.07 | 0.02 | -3.26 | **0.003** |
| NONE - LEX | REC | 0.08 | 0.02 | 3.71 | **<0.001** |
| NONE - MATH | REC | 0.05 | 0.02 | 2.57 | **0.020** |
| VIS - LEX | REC | 0.15 | 0.02 | 6.95 | **<0.001** |
| VIS - MATH | REC | 0.12 | 0.02 | 5.84 | **<0.001** |
| LEX - MATH | REC | -0.02 | 0.02 | -1.16 | 0.245 |

**Table S2. Pairwise differences for the model RT = condition × task + (1|ID). Primary tasks:** REC: Visual Recursion Task; ITE: Visual Iteration Task; **Secondary tasks:** VIS: Visual interference, LEX: lexical retrieval, MATH: serial arithmetics; NONE: no interference.

|  | **Acc bin** | | | **log RT** | | |
| --- | --- | --- | --- | --- | --- | --- |
| *Predictors* | *Odds Ratios* | *CI* | *p* | *Estimates* | *CI* | *p* |
| (Intercept) | 6.57 | 4.18 – 10.33 | **<0.001** | 0.64 | 0.57 – 0.71 | **<0.001** |
| Task [REC-ITE] | 5.39 | 2.17 – 13.37 | **<0.001** | -0.15 | -0.24 – -0.06 | **0.002** |
| Condition [VIS-NONE] | 0.96 | 0.54 – 1.70 | 0.882 | 0.15 | 0.08 – 0.23 | **<0.001** |
| Condition [LEX-NONE] | 0.38 | 0.23 – 0.64 | **<0.001** | 0.10 | 0.03 – 0.17 | **0.006** |
| Condition [MATH-NONE] | 0.54 | 0.32 – 0.92 | **0.023** | 0.08 | 0.01 – 0.15 | **0.030** |
| Foil [ODD-REP] | 1.49 | 0.80 – 2.77 | 0.206 | 0.03 | -0.04 – 0.10 | 0.420 |
| Foil [POS-REP] | 1.15 | 0.64 – 2.07 | 0.648 | -0.11 | -0.19 – -0.04 | **0.002** |
| [REC-ITE] ×[VIS-NONE] | 0.36 | 0.12 – 1.06 | 0.065 | -0.03 | -0.13 – 0.07 | 0.607 |
| [REC-ITE] × [LEX-NONE] | 0.37 | 0.14 – 0.99 | **0.047** | -0.09 | -0.19 – 0.01 | 0.067 |
| [REC-ITE] × [MATH-NONE] | 0.27 | 0.10 – 0.73 | **0.010** | -0.04 | -0.14 – 0.06 | 0.399 |
| [REC-ITE] × [ODD-REP] | 0.05 | 0.02 – 0.14 | **<0.001** | 0.27 | 0.17 – 0.37 | **<0.001** |
| [REC-ITE] × [POS-REP] | 0.13 | 0.05 – 0.36 | **<0.001** | 0.32 | 0.22 – 0.42 | **<0.001** |
| [VIS-NONE] × [ODD-REP] | 0.59 | 0.26 – 1.36 | 0.219 | -0.08 | -0.18 – 0.02 | 0.129 |
| [LEX-NONE] × [ODD-REP] | 0.69 | 0.32 – 1.48 | 0.341 | 0.01 | -0.10 – 0.11 | 0.920 |
| [MATH-NONE] × [ODD-REP] | 0.33 | 0.15 – 0.72 | **0.005** | 0.05 | -0.05 – 0.16 | 0.295 |
| [VIS-NONE] × [POS-REP] | 0.66 | 0.30 – 1.48 | 0.316 | 0.07 | -0.03 – 0.17 | 0.174 |
| [LEX-NONE] × [POS-REP] | 0.85 | 0.40 – 1.78 | 0.665 | 0.14 | 0.04 – 0.24 | **0.006** |
| [MATH-NONE] × [POS-REP] | 0.59 | 0.28 – 1.24 | 0.164 | 0.14 | 0.04 – 0.25 | **0.005** |
| [R-I] × [VIS-NO] × [ODD-REP] | 11.93 | 3.13 – 45.44 | **<0.001** | -0.04 | -0.19 – 0.10 | 0.567 |
| [R-I] × [LEX-NO] × [ODD-REP] | 9.80 | 2.86 – 33.60 | **<0.001** | -0.20 | -0.34 – -0.06 | **0.007** |
| [R-I] × [MATH-NO] × [ODD-REP] | 29.35 | 8.47 – 101.71 | **<0.001** | -0.28 | -0.42 – -0.13 | **<0.001** |
| [R-I] × [VIS-NO] × [POS-REP] | 4.33 | 1.15 – 16.30 | **0.030** | -0.13 | -0.27 – 0.01 | 0.078 |
| [R-I] × [LEX-NO] × [POS-REP] | 5.58 | 1.62 – 19.22 | **0.006** | -0.22 | -0.37 – -0.08 | **0.002** |
| [R-I] × [MATH-NO] × [POS-REP] | 8.03 | 2.31 – 27.85 | **0.001** | -0.21 | -0.35 – -0.07 | **0.004** |

**Table S3. Pairwise differences for the models: accuracy = condition × task × foil + (1|ID) and rt = condition × task × foil + (1|ID). Primary tasks: [**R]EC: Visual Recursion Task; [I]TE: Visual Iteration Task; **Secondary tasks:** VIS: Visual interference, LEX: lexical retrieval, MATH: serial arithmetics; NONE: no interference. **Foil categories:** ODD: odd foils; REP: repeat foils; POS: positional foils.

**Figure S1. Accuracy differences between tasks and conditions across foils.**

**Figure S2. RT differences between tasks and conditions across foils.**

**
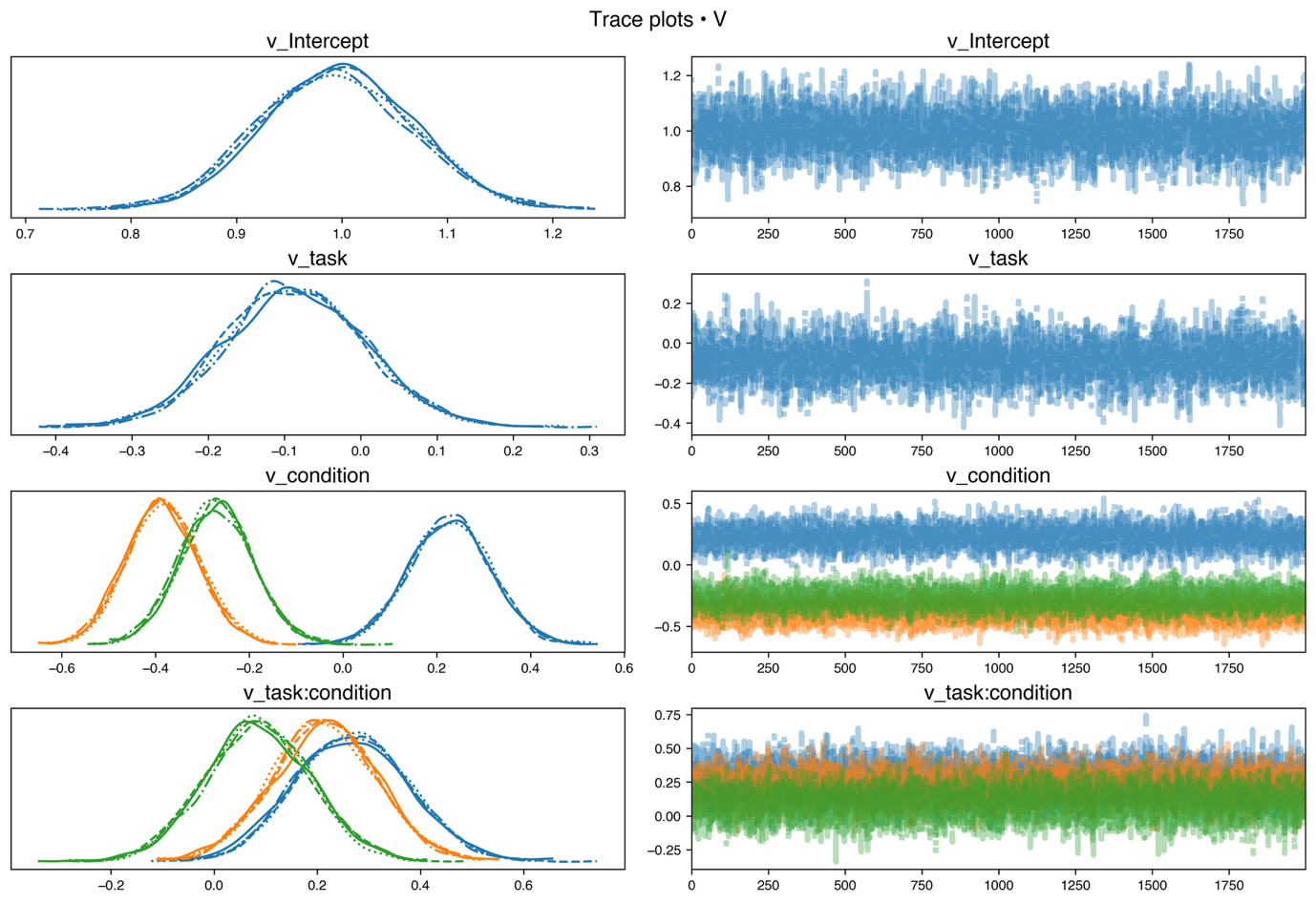
**

**Figure S3. Trace plots for drift rate parameters (*v*).** Posterior draws (left) and kernel density estimates (right) for drift rate intercept, task, condition, and task × condition interaction terms. Each panel shows four independent MCMC chains. Visual inspection indicates satisfactory convergence (no major drifts across chains, stable posterior densities).

**
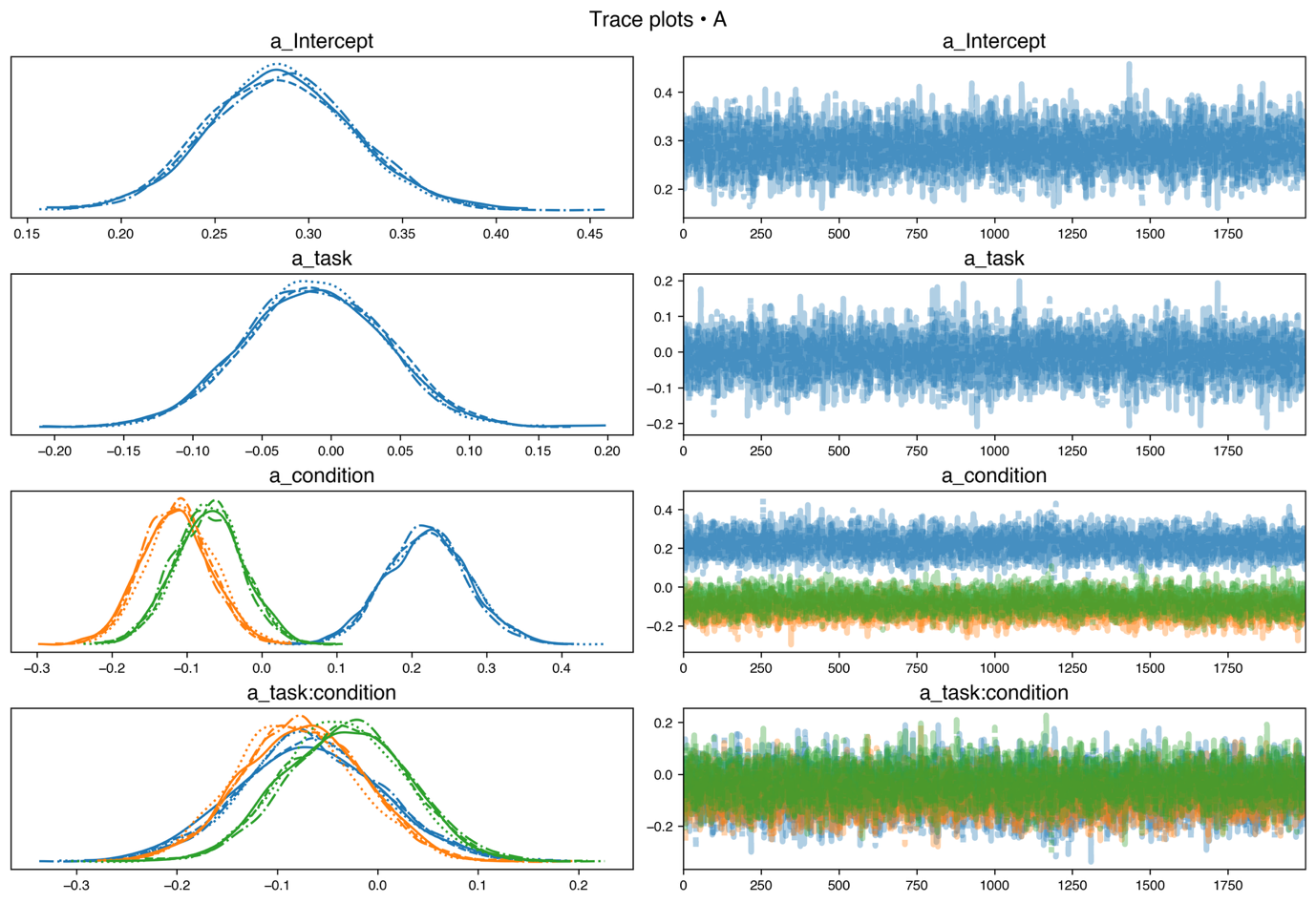
**

**Figure S4.** **Trace plots for boundary separation parameters (*a*).** Posterior draws (left) and kernel density estimates (right) for drift rate intercept, task, condition, and task × condition interaction terms. Each panel shows four independent MCMC chains. Chains show good mixing, with overlapping distributions across chains.

**
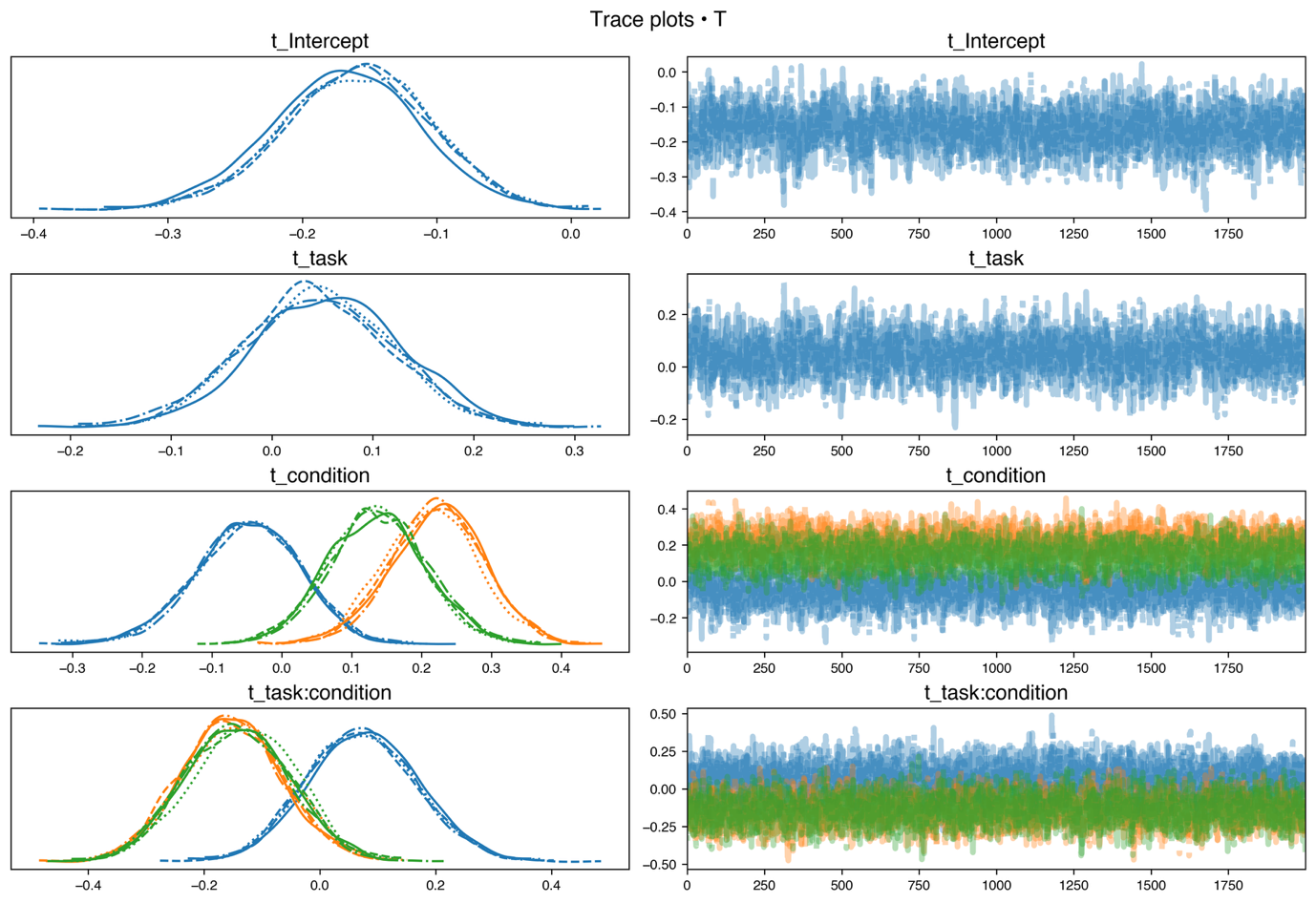
**

**Figure S5.** **Trace plots for non-decision time parameters (*t*).** Posterior draws (left) and kernel density estimates (right) for drift rate intercept, task, condition, and task × condition interaction terms. Each panel shows four independent MCMC chains. Trace plots confirm stable sampling with no evidence of chain divergence.

| **contrast** | **M** | **HDI95** | **P(>0)** | **evidence** |
| --- | --- | --- | --- | --- |
| v: ITE VIS | 0.228 | [0.058, 0.394] | 0.995 | ★★ |
| v: ITE LEX | -0.385 | [-0.540, -0.240] | 0.000 | ★★ |
| v: ITE MATH | -0.270 | [-0.421, -0.116] | 0.000 | ★★ |
| v: REC VIS | 0.495 | [0.319, 0.683] | 1.000 | ★★ |
| v: REC LEX | -0.172 | [-0.344, -0.007] | 0.023 | ★★ |
| v: REC MATH | -0.185 | [-0.356, -0.020] | 0.016 | ★★ |
| v: (REC–ITE) VIS | 0.267 | [0.060, 0.484] | 0.993 | ★★ |
| v: (REC–ITE) LEX | 0.213 | [0.016, 0.412] | 0.982 | ★★ |
| v: (REC–ITE) MATH | 0.085 | [-0.112, 0.286] | 0.799 |  |
|  |  |  |  |  |
| a: ITE VIS | 0.330 | [0.161, 0.503] | 1.000 | ★★ |
| a: ITE LEX | -0.143 | [-0.250, -0.029] | 0.007 | ★★ |
| a: ITE MATH | -0.091 | [-0.207, 0.023] | 0.059 |  |
| a: REC VIS | 0.220 | [0.055, 0.370] | 0.998 | ★★ |
| a: REC LEX | -0.228 | [-0.332, -0.123] | 0.000 | ★★ |
| a: REC MATH | -0.130 | [-0.239, -0.011] | 0.012 | ★★ |
| a: (REC–ITE) VIS | -0.111 | [-0.326, 0.105] | 0.154 |  |
| a: (REC–ITE) LEX | -0.085 | [-0.232, 0.058] | 0.131 |  |
| a: (REC–ITE) MATH | -0.038 | [-0.193, 0.107] | 0.313 |  |
|  |  |  |  |  |
| t: ITE VIS | -0.039 | [-0.157, 0.081] | 0.263 |  |
| t: ITE LEX | 0.211 | [0.079, 0.350] | 0.999 | ★★ |
| t: ITE MATH | 0.125 | [0.001, 0.261] | 0.973 | ★★ |
| t: REC VIS | 0.022 | [-0.106, 0.147] | 0.634 |  |
| t: REC LEX | 0.065 | [-0.056, 0.185] | 0.853 |  |
| t: REC MATH | -0.002 | [-0.124, 0.119] | 0.477 |  |
| t: (REC–ITE) VIS | 0.062 | [-0.101, 0.217] | 0.771 |  |
| t: (REC–ITE) LEX | -0.146 | [-0.322, 0.016] | 0.046 | ★ |
| t: (REC–ITE) MATH | -0.127 | [-0.293, 0.030] | 0.060 |  |

**Table S4. Posterior contrasts from the hierarchical drift diffusion model (HDDM).** Reported are mean posterior estimates (M), 95% highest density intervals (HDI95), posterior probabilities (P(>0)), and evidence strength (★ = moderate, ★★ = strong/very strong). Parameters: drift rate (v = speed of evidence accumulation), boundary separation (a = amount of evidence required for a decision), and non-decision time (t = perceptual/motor latency). Primary tasks: REC = Visual Recursion Task; ITE = Visual Iteration Task. Secondary tasks: VIS = visual interference; LEX = lexical retrieval; MATH = serial arithmetics; NONE = no interference. All contrasts are expressed relative to the baseline condition (NONE) within task, or as task differences (REC–ITE) within condition.

**
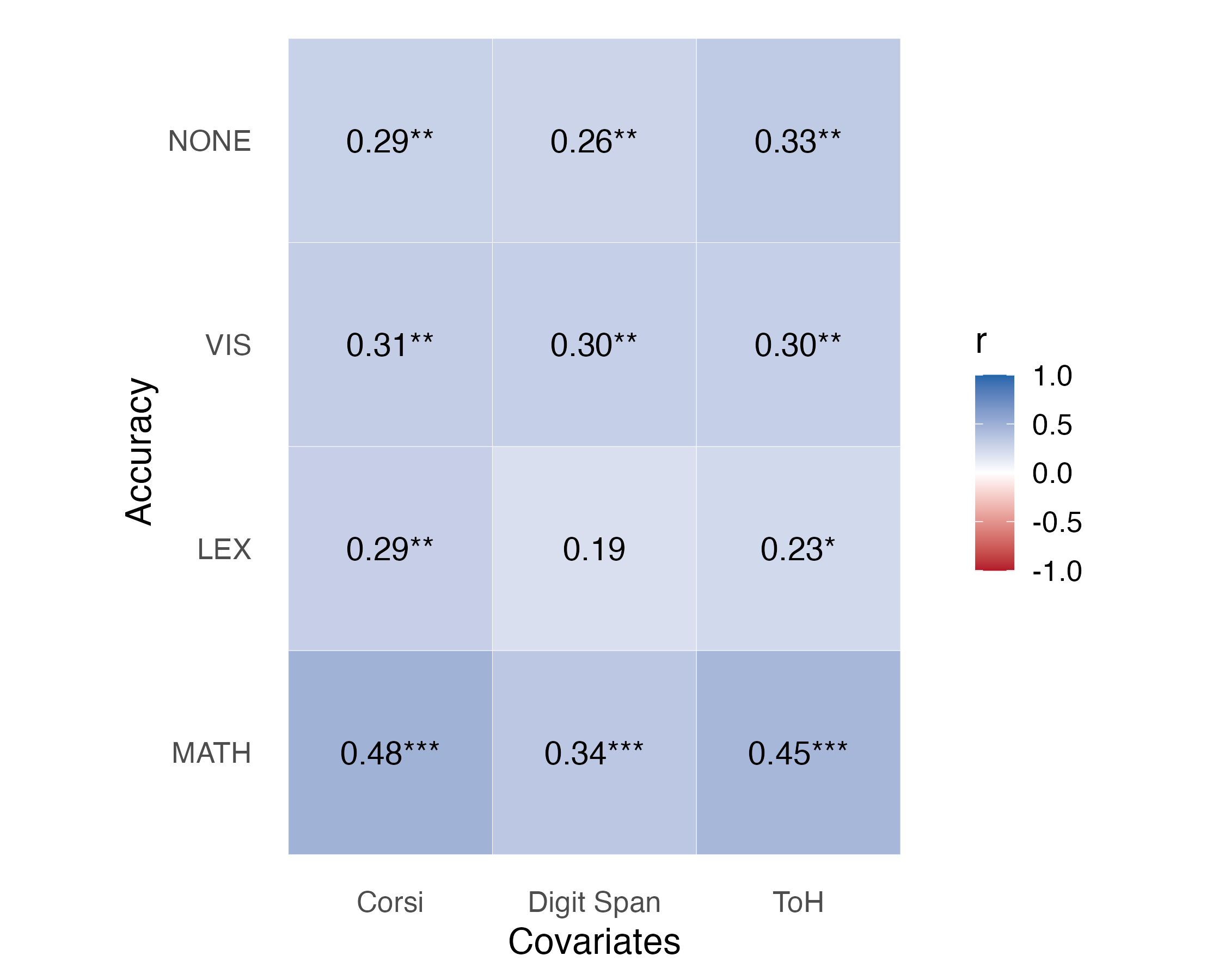
**

**Figure S6. Pearson’s correlations between accuracy in the REC/ITE task conditions and cognitive correlates.** NONE = no interference; VIS = visual interference; LEX = lexical retrieval; MATH = serial arithmetics; Corsi = Corsi block tapping (spatial working memory); Digit Span (verbal working memory task); ToH = Tower of Hanoi (Hierarchical planning task).

| **Accuracy** | | | | |
| --- | --- | --- | --- | --- |
|  | | | | |
|  | Dependent variable: | | | |
|  |  | | | |
|  | NONE | VIS | LEX | MATH |
|  | (1) | (2) | (3) | (4) |
|  | | | | |
| corsi | 0.039 (0.138) | 0.173 (0.125) | 0.207 (0.153) | 0.235^*^ (0.134) |
| dspan | -0.101 (0.130) | -0.018 (0.123) | 0.086 (0.143) | 0.043 (0.126) |
| toh | -0.137 (0.221) | -0.202 (0.206) | 0.246 (0.245) | 0.110 (0.221) |
| REC-ITE | -0.043 (0.183) | -0.064 (0.171) | 0.033 (0.204) | 0.047 (0.180) |
| rt NONE | -0.249^**^ (0.095) |  |  |  |
| rt VIS |  | -0.396^***^ (0.088) |  |  |
| rt LEX |  |  | -0.306^***^ (0.104) |  |
| rt MATH |  |  |  | -0.267^***^ (0.089) |
| REC-ITE:corsi | -0.025 (0.213) | -0.003 (0.197) | 0.057 (0.247) | 0.039 (0.205) |
| REC-ITE:dpsan | 0.394^**^ (0.188) | 0.134 (0.172) | 0.190 (0.209) | 0.304^*^ (0.182) |
| REC-ITE:toh | 0.228 (0.306) | 0.160 (0.281) | -0.327 (0.338) | 0.166 (0.304) |
| Constant | 0.297^**^ (0.134) | 0.309^**^ (0.123) | 0.136 (0.148) | 0.137 (0.131) |
|  | | | | |
| Observations | 90 | 90 | 91 | 91 |
| R^2^ | 0.155 | 0.268 | 0.180 | 0.316 |
| Adjusted R^2^ | 0.072 | 0.196 | 0.100 | 0.249 |
|  | | | | |
| Note: | ^*^p^**^p^***^p<0.01 | | | |

**Table S5. Linear models predicting accuracy in baseline (NONE) and interference conditions (VIS, LEX, MATH) from the cognitive covariates (Corsi, Digit span, Tower of Hanoi), task (REC vs. ITE), RT, and their interactions, with specific focus on the task × covariate interaction terms.** NONE = no interference; VIS = visual interference; LEX = lexical retrieval; MATH = serial arithmetics; Corsi = Corsi block tapping (spatial working memory); Digit Span (verbal working memory task); ToH = Tower of Hanoi (Hierarchical planning task).

| DV | Effect | Power | CI_low | CI_high |
| --- | --- | --- | --- | --- |
| Accuracy | (REC-ITE)×(LEX-NONE) | 0.79 | 0.77 | 0.82 |
| Accuracy | (REC-ITE)×(MATH-NONE) | 0.97 | 0.96 | 0.98 |
| Accuracy | (REC-ITE)×(VIS-NONE) | 0.68 | 0.65 | 0.71 |
| RT | (REC-ITE)×(LEX-NONE) | 1 | 0.99 | 1 |
| RT | (REC-ITE)×(MATH-NONE) | 1 | 0.99 | 1 |
| RT | (REC-ITE)×(VIS-NONE) | 0.79 | 0.76 | 0.81 |

**Table S6.** **Retrospective statistical power for key Task × Condition interaction terms, based on 1,000 parametric simulations from the fitted mixed-effects models.** Power was estimated separately for accuracy (logistic mixed model: Acc_bin ~ Task * Condition + (1 | ID)) and reaction times (linear mixed model: logRT ~ Task * Condition + (1 | ID)). The reference levels were *ITE* for **Task** and *NONE* for **Condition**, so each interaction contrast reflects interference-related change relative to baseline ITE performance. For each contrast, power values reflect the proportion of simulated datasets in which the corresponding interaction term (e.g., (REC-ITE)×(LEX-NONE)) reached statistical significance under the observed effect size. Simulations used the *simr* package with 1,000 iterations per contrast.

**
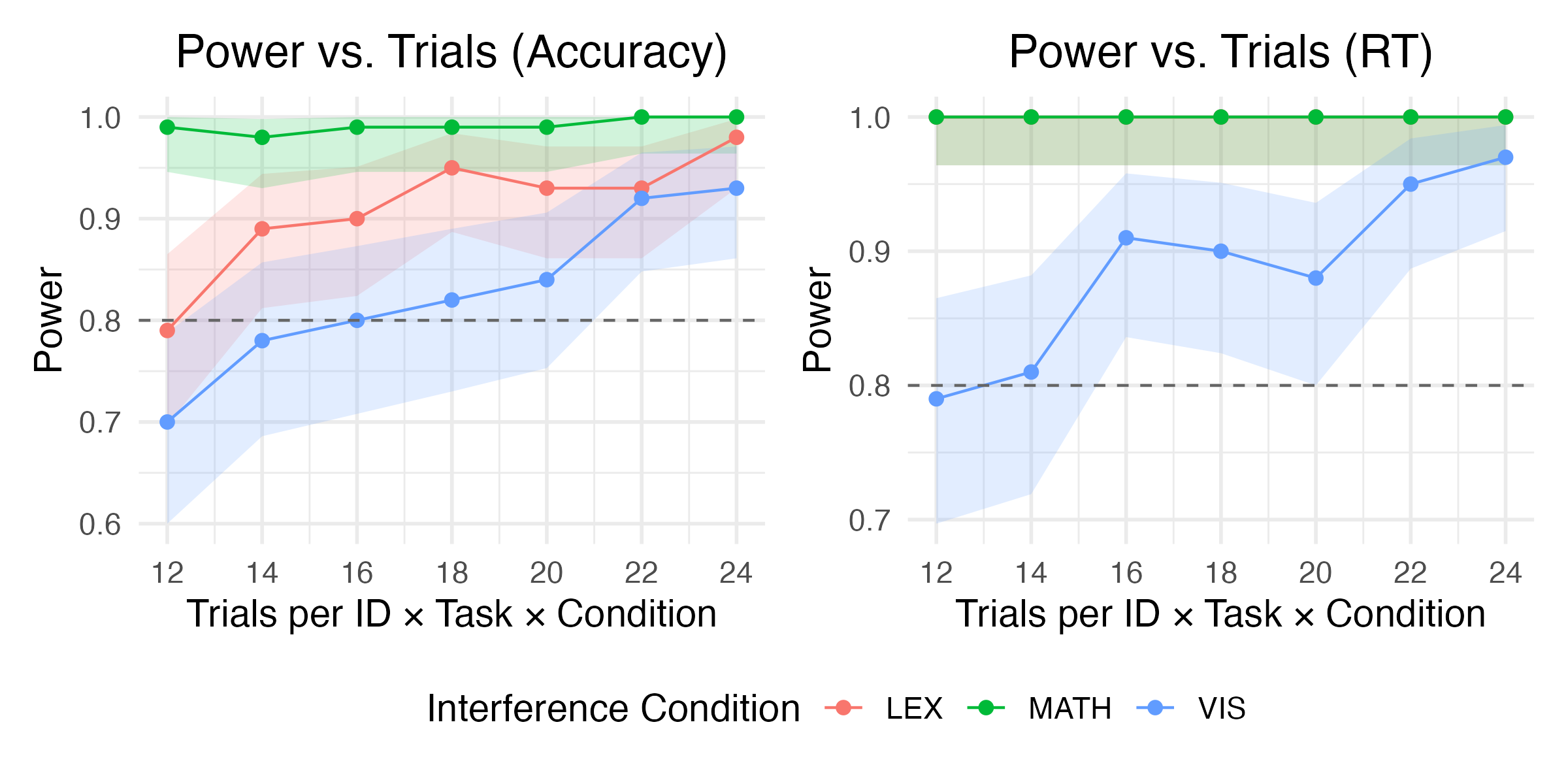
**

**Figure S7. Power simulations across interference conditions (100 iterations).** We simulated the power of the accuracy (left) and response-time (right) models by increasing the number of trials from 12 to 24 per participant, task, and condition. Colored ribbons depict 95% confidence intervals.

**
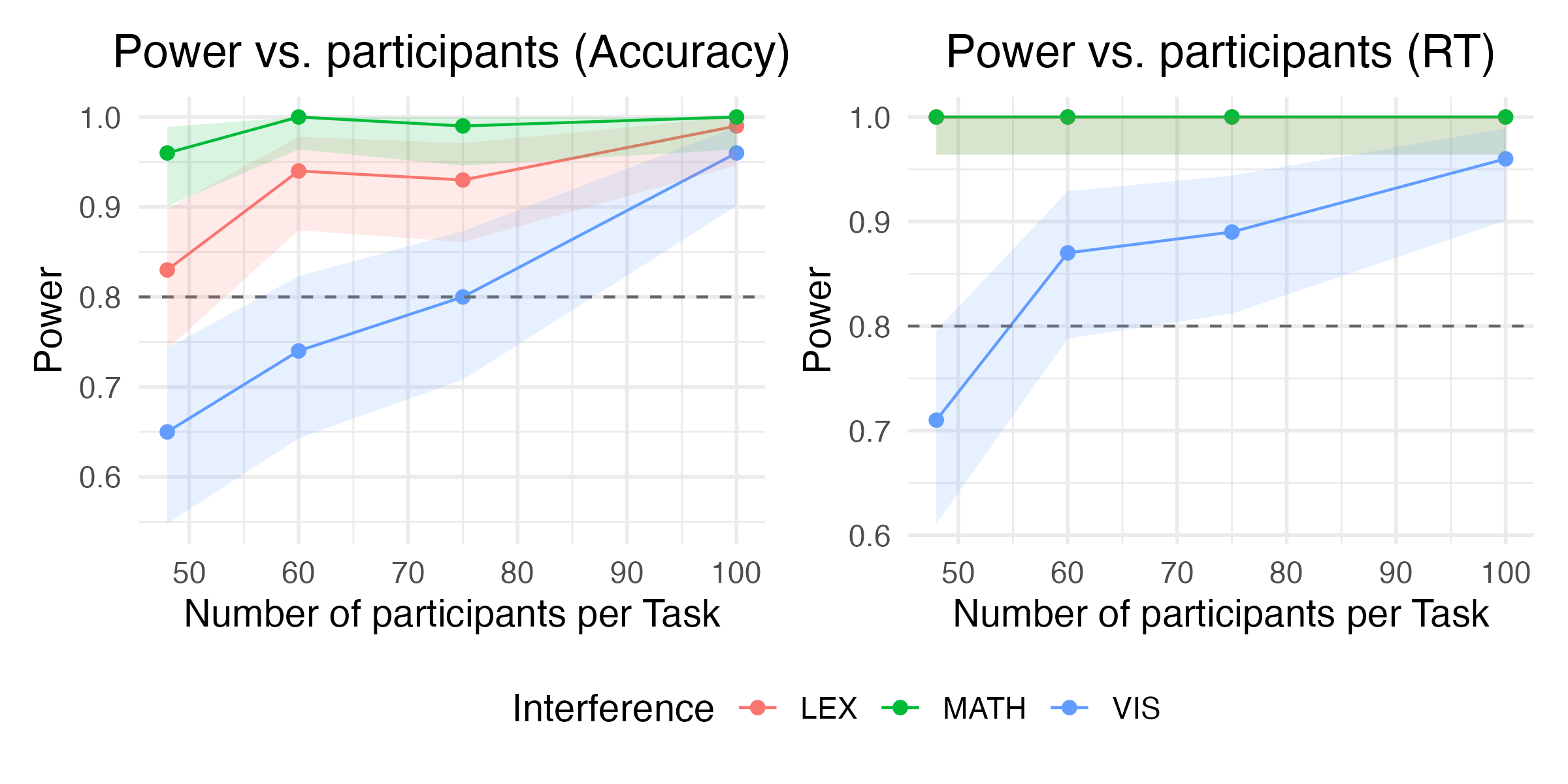
**

**Figure S8. Power simulations across interference conditions (100 iterations).** We simulated the power of the accuracy (left) and response-time (right) models by increasing the number of participants from 48 to 100. Colored ribbons depict 95% confidence intervals.
